# The *Mycobacterium tuberculosis* ESX-5 secretion system enables carbon source utilization and growth in mice

**DOI:** 10.1128/mbio.03500-25

**Published:** 2026-01-30

**Authors:** Alisha M. Block, Rashmi Ravindran Nair, Virginia Meikle, Parker C. Wiegert, Dylan W. White, Leanne Zhang, Michael Niederweis, Anna D. Tischler

**Affiliations:** 1 Department of Microbiology and Immunology, University of Minnesota 205104, Minneapolis, Minnesota, USA; 2 Department of Microbiology, University of Alabama at Birmingham 318277, Birmingham, Alabama, USA; The Hebrew University of Jerusalem, Rehobot, Israel

**Keywords:** type VII secretion, outer membrane proteins, glucose transport, ESX-5, nutrient transport, *Mycobacterium tuberculosis*, heavy metals

## Abstract

*Mycobacterium tuberculosis* uses several ESX type VII protein secretion systems for pathogenesis. *M. tuberculosis* ESX-5 is only partially characterized because it is essential for growth in standard lab culture conditions. To circumvent ESX-5 essentiality, we made an *M. tuberculosis* strain in which the central ESX-5 membrane component EccD_5_ can be conditionally depleted. Here, we use this strain to demonstrate that *M. tuberculosis* requires the ESX-5 secretion system to grow using specific carbon sources *in vitro*, to grow in cultured macrophages, and to replicate and disseminate in aerosol-infected mice. *M. tuberculosis* requires ESX-5 to use glycerol or glucose as the sole carbon source. Use of glycerol and glucose also depends on the outer membrane protein PPE51. We show that *M. tuberculosis* requires ESX-5 activity for outer membrane export and surface exposure of PPE51. Expression of the outer membrane porin MspA enabled growth of ESX-5-deficient *M. tuberculosis* on glycerol, suggesting that the main function of ESX-5 *in vitro* is to export nutrient transporters to the outer membrane. Depletion of EccD_5_ in acutely infected mice caused clearance of *M. tuberculosis* from lung tissues, demonstrating the critical importance of ESX-5 activity during infection. Our findings suggest that ESX-5 promotes *M. tuberculosis* pathogenesis by mediating export of outer membrane proteins that enable nutrient acquisition.

**IMPORTANCE:**

*Mycobacterium tuberculosis* ESX type VII secretion systems play important roles in pathogenesis, but the functions of ESX-5 are not well characterized because it is essential for growth in standard lab culture conditions. We used a strain that conditionally expresses a central membrane component of the ESX-5 secretion apparatus to determine how ESX-5 impacts growth in lab cultures and in a mouse infection model. We found that *M. tuberculosis* requires ESX-5 to grow using several carbon sources and to grow in the lungs of infected mice. Inhibiting production of the ESX-5 secretion system in mice also led to clearance of *M. tuberculosis* from lung tissues. Our results demonstrate that the *M. tuberculosis* ESX-5 system is a critical virulence factor and suggest that ESX-5 is a strong candidate for antitubercular drug development.

## INTRODUCTION

*Mycobacterium tuberculosis* encodes five ESX type VII protein secretion systems that play critical roles in pathogenesis by exporting proteins that manipulate the functions of infected phagocytes (1, 2). ESX-1, ESX-2, and ESX-4 all participate in permeabilizing the phagosomal membrane, enabling *M. tuberculosis* and secreted molecules to access the host cell cytosol (3, 4). ESX-1 induces pyroptosis of infected macrophages by disrupting the plasma membrane, which activates the NLRP3 inflammasome (5–7). ESX-4 exports the tuberculosis necrotizing toxin (CpnT or TNT) that triggers macrophage necrosis (3, 8). *M. tuberculosis* requires ESX-3 to acquire iron (9, 10) and to prevent ESCRT-dependent phagosomal membrane repair, which inhibits antigen processing and T-cell activation (11, 12). The related pathogen *Mycobacterium marinum* requires both ESX-5 and ESX-1 to induce inflammasome activation and pyroptotic death of infected macrophages (13). However, analysis of ESX-5 function in *M. tuberculosis* pathogenesis has been complicated by the fact that ESX-5 is essential for *in vitro* growth.

Genetic evidence suggests that *M. tuberculosis* requires ESX-5 for growth in standard lab culture conditions. Genes encoding ESX-5 conserved components that form an inner membrane secretion complex (EccB_5_, EccC_5_, EccD_5_, EccE_5_, and MycP_5_ [14, 15]) are essential for growth of *M. tuberculosis* lab strains and clinical isolates in standard culture media based on genome-wide Tn-seq screens (16, 17). The *eccB_5_*, *eccC_5_*, and *eccD_5_* genes were confirmed to be essential as these genes could not be deleted without a complementing copy provided in *trans* (18, 19). Both *eccC_5_* and *mycP_5_* are similarly essential in *M. marinum* (20). *Mycobacterium bovis* BCG also requires *mycP_5_* for *in vitro* growth (20). *M. tuberculosis* ∆*eccD_5_* and ∆*esx-5* mutants, in which the entire *esx-5* locus is deleted, are described (21–23). However, one ∆*esx-5* strain has a secondary mutation that prevents production of phthiocerol dimycocerosate (PDIM) (23), an outer membrane lipid essential for virulence (24, 25). Rigorous genetic analyses were not done to confirm the absence of compensatory mutations in the other ∆*esx-5* mutants or the ∆*eccD_5_* mutant (21, 22). The ∆*eccD_5_* mutant failed to replicate in intravenously infected severe combined immunodeficiency (SCID) mice, but this phenotype was not fully complemented (21). A ∆*esx-5* mutant similarly failed to replicate in intravenously infected SCID or *Rag*^−/−^ mice (22), but complementation was not done. Thus, ESX-5 likely plays a critical role in *M. tuberculosis* physiology that promotes growth in the host, but this has yet to be tested directly in an immune-competent animal model.

Clues to the function of ESX-5 in mycobacterial physiology were provided by studies in *M. marinum*, which suggested that ESX-5 enables *in vitro* growth by increasing permeability of the mycobacterial outer membrane to nutrients (20). The *eccC_5_* or *mycP_5_* genes could be deleted in *M. marinum* expressing the *Mycobacterium smegmatis* porin MspA (20), which transports small hydrophilic nutrients, including glucose and serine, across the *M. smegmatis* outer membrane (26, 27). In *M. marinum*, *eccC_5_* could also be disrupted in strains lacking PDIM (20). The PDIM lipid decreases permeability of the mycobacterial outer membrane to small molecules, including nutrients and antibiotics (28–31). Taken together, these data imply that ESX-5 normally functions to export proteins that enhance nutrient transport through the *M. marinum* outer membrane.

Based on studies done in *M. marinum*, ESX-5 exports most proteins of the mycobacteria-specific PE and PPE families (20, 32, 33). The *M. tuberculosis* H37Rv reference genome encodes 99 PE proteins and 69 PPE proteins, representing ~8% of the genome coding capacity (34, 35). Most of these *M. tuberculosis* PE and PPE proteins are likely to be exported via ESX-5, as immune recognition of conserved PE and PPE epitopes requires a functional ESX-5 secretion system (36). A subset of PE and PPE proteins was implicated in the uptake of small molecule nutrients, including heme, Ca^2+^, glucose, glycerol, and various disaccharides (28, 37–43). Some PPE proteins linked to nutrient uptake localize to the *M. tuberculosis* cell surface where they have been proposed to act either as receptors that capture nutrients or as porin-like channels that facilitate nutrient transport through the outer membrane (28, 37, 41). A major function of ESX-5 that promotes *M. tuberculosis* growth *in vitro* may be to export PE and PPE proteins to enable nutrient acquisition. However, the molecular mechanisms by which ESX-5 promotes *in vitro* growth and the role of ESX-5 in *M. tuberculosis* pathogenesis have not been determined.

Here we use a *M. tuberculosis* strain that conditionally expresses the ESX-5 core component EccD_5_ to determine the roles of ESX-5 in growth *in vitro*, in macrophages, and in mice. We find that EccD_5_ depletion prevents growth on glucose and glycerol, the primary carbon sources in standard lab media. We demonstrate that *M. tuberculosis* requires ESX-5 for outer membrane localization of PPE51, which was previously implicated in glucose and glycerol uptake (28), suggesting that ESX-5 exports PPE51 to enable growth on glucose and glycerol. In macrophages, we find that *M. tuberculosis* requires ESX-5 for replication and for induction of inflammatory cell death. Finally, in mice, we find that EccD_5_ depletion prevents *M. tuberculosis* replication in the lungs and dissemination to the spleen and causes clearance of the bacteria during acute infection. Collectively, our findings suggest that ESX-5 promotes *M. tuberculosis* pathogenesis in part by exporting outer membrane proteins that enable nutrient acquisition.

## RESULTS

To circumvent the essentiality of ESX-5 for *M. tuberculosis* growth *in vitro*, we generated an *eccD_5_* Tet-OFF strain in which the native *eccD_5_* gene is deleted and *eccD_5_* is expressed in *trans* from a tetracycline-repressible (Tet-OFF) promoter (19). We showed that *eccD_5_* is transcriptionally repressed; EccD_5_ protein is depleted, and secretion of two known ESX-5 substrates (EsxN and PPE41) is reduced upon treatment of *eccD_5_* Tet-OFF with anhydrotetracycline (Atc) (3, 19). We verified by whole genome sequencing that our *eccD_5_* Tet-OFF strain does not harbor mutations in genes required to produce the PDIM lipid. We also confirmed by analysis of ^14^C-propionate-labeled lipid extracts that *eccD_5_* Tet-OFF produces PDIM (Fig. S1A). We now exploit this *eccD_5_* Tet-OFF strain to determine the role of the ESX-5 secretion system in *M. tuberculosis* growth *in vitro* and in mice.

### *M. tuberculosis* requires ESX-5 to grow on specific carbon sources *in vitro*

**Fig 1:**
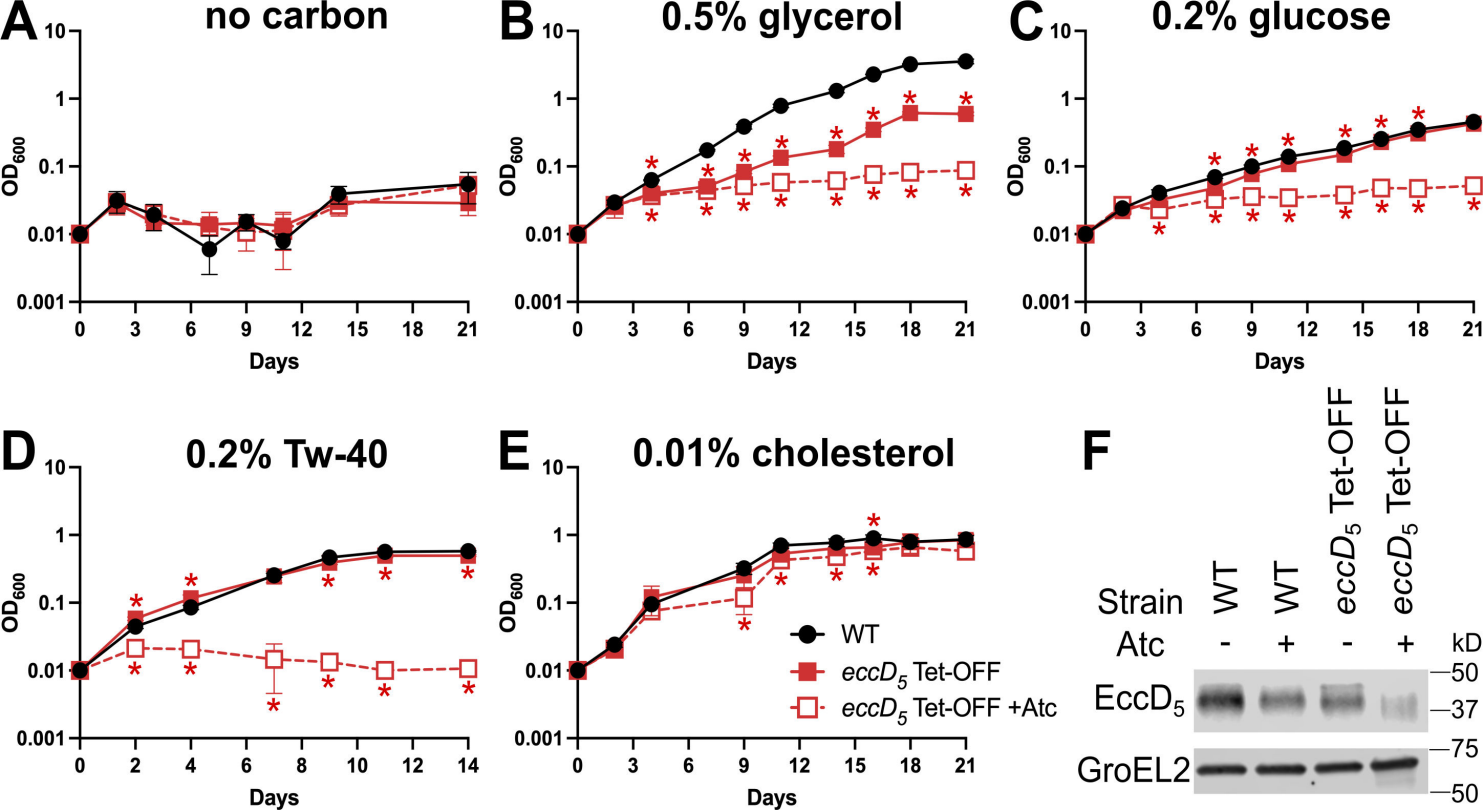
*M. tuberculosis* requires ESX-5 for *in vitro* growth on various carbon sources. (**A–E**) WT Erdman and *eccD_5_* Tet-OFF were grown in complete Middlebrook 7H9 ± 100 ng/mL anhydrotetracycline (Atc) to mid-exponential phase, then washed and diluted to OD_600_ = 0.01 in triplicate in Middlebrook 7H9 base with 0.01% tyloxapol and no added carbon (**A**), 0.5% glycerol (**B**), 0.2% glucose (**C**), 0.2% Tween-40 (**D**), or 0.01% cholesterol (**E**) ± 100 ng/mL Atc. In panel E, fresh cholesterol was added at 0.01% every 2–3 days. Growth was monitored by OD_600_ measurements every 2–3 days. Fresh Atc (100 ng/mL) was added to +Atc cultures every 7 days. Data are means ± standard deviations. A one-way ANOVA with Dunnett’s correction was used to compare *eccD_5_* Tet-OFF ± Atc to WT (*P < 0.05). Complete results of statistical analyses are in Table S4. (**F**) Proteins were extracted from cholesterol cultures at day 21. EccD_5_ and GroEL2 were detected in whole cell lysates (11.6 μg total protein) by Western blotting.

Previous studies in *M. marinum* implicated ESX-5 in growth on esterified fatty acids (20). *M. marinum* also likely requires ESX-5 for growth on small hydrophilic carbon sources, as ESX-5 core components could be deleted in a strain expressing the *M. smegmatis* porin MspA (20, 26, 27). To determine the role of *M. tuberculosis* ESX-5 in carbon source utilization, we analyzed the growth of our *eccD_5_* Tet-OFF strain in Middlebrook 7H9 base medium supplemented with various sole carbon sources. The 7H9 base does not support growth of wild-type (WT) *M. tuberculosis* Erdman or *eccD_5_* Tet-OFF (Fig. 1A). WT Erdman and *eccD_5_* Tet-OFF grew on all carbon sources tested (Fig. 1B through E), though growth of the *eccD_5_* Tet-OFF strain was significantly delayed on glycerol, even without Atc (Fig. 1B). Depletion of EccD_5_ with Atc prevented *M. tuberculosis* growth on glycerol (Fig. 1B), glucose (Fig. 1C), and the esterified fatty acid Tween-40 (Tw-40, Fig. 1D). We observed similar growth defects on glycerol or glucose in a minimal salt medium when EccD_5_ was depleted (Fig. S2). In contrast, EccD_5_ depletion only slightly reduced the growth rate on cholesterol as the sole carbon source (Fig. 1E), suggesting that *M. tuberculosis* does not require ESX-5 to use cholesterol or other nutrients in Middlebrook 7H9. We confirmed that EccD_5_ was efficiently depleted from the *eccD_5_* Tet-OFF strain grown in 7H9 with cholesterol + Atc (Fig. 1F). These data indicate that *M. tuberculosis* requires ESX-5 to grow using glycerol, glucose, or Tw-40 as the sole carbon source. As glycerol and glucose are the primary carbon sources in Middlebrook media, our data suggest that ESX-5 is essential *in vitro* because it is required to use these carbon sources.

### *M. tuberculosis* requires ESX-5 for nutrient uptake through the mycobacterial outer membrane

**Fig 2:**
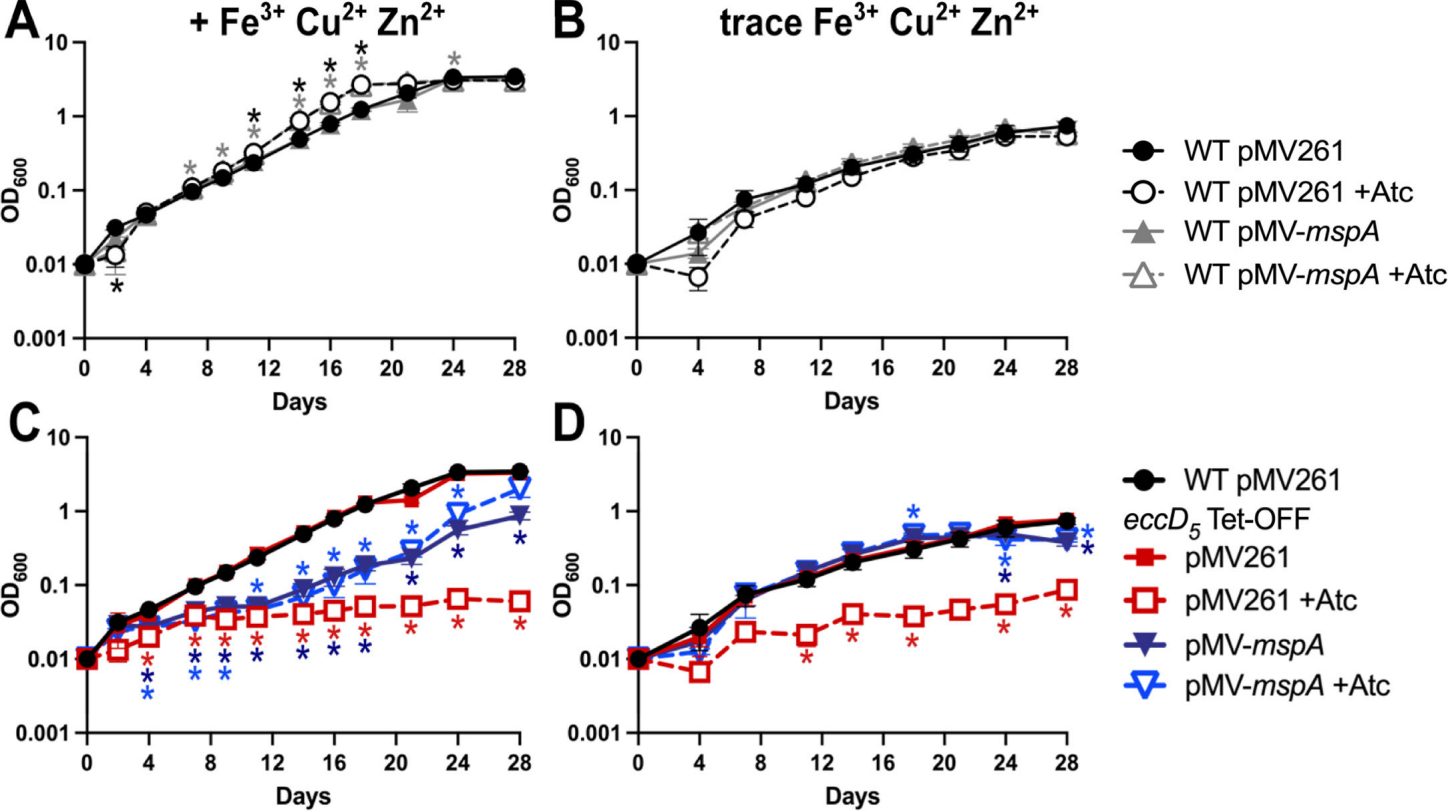
Expression of the *M. smegmatis* porin MspA rescues growth of EccD_5_-deficient *M. tuberculosis* on glycerol. The indicated strains were grown in complete Middlebrook 7H9 ± 100 ng/mL anhydrotetracycline (Atc) to mid-exponential phase, then washed and diluted in triplicate to obtain OD_600_ = 0.01 in Middlebrook 7H9 base with 0.01% tyloxapol and 0.5% glycerol (**A and C**) or self-made trace heavy metal Middlebrook 7H9 (Fe^3+^, Cu^2+^, and Zn^2+^ omitted) with 0.01% tyloxapol and 0.5% glycerol (**B and D**) ± 100 ng/mL Atc. Growth was monitored by OD_600_ measurements every 2–3 days. Fresh Atc (100 ng/mL) was added to +Atc cultures every 7 days. Data are means ± standard deviations. Statistical analysis was done to compare each strain and condition to the WT pMV261 untreated control (**A and B**) or to compare each strain and condition to the *eccD_5_* Tet-OFF pMV261 untreated control (**C and D**) (*P < 0.05, two-way ANOVA with a simple effect model and Dunnett’s correction). Complete results of statistical analyses are shown in Table S4.

Loss of ESX-5 function in *M. marinum* could be complemented by expression of the MspA outer membrane porin from *M. smegmatis* (20). We similarly attempted to complement *in vitro* growth of our *eccD_5_* Tet-OFF strain with MspA. Expression of MspA in *trans* from pMV-*mspA* did not affect the growth of WT *M. tuberculosis* (Fig. 2A) but significantly improved the growth of EccD_5_-depleted *M. tuberculosis* in 7H9 with glycerol as the sole carbon source (Fig. 2C). Western blotting confirmed that EccD_5_ was depleted by Atc in the *eccD_5_* Tet-OFF pMV-*mspA* strain (Fig. S3A). The *eccD_5_* Tet-OFF pMV261 and *eccD_5_* Tet-OFF pMV-*mspA* strains both produce PDIM (Fig. S1B), so growth of the MspA-expressing strain on glycerol is not due to loss of the PDIM lipid. However, even without Atc, we observed an extended lag phase for the *eccD_5_* Tet-OFF pMV-*mspA* strain relative to the *eccD_5_* Tet-OFF pMV261 and WT controls (Fig. 2C).

In addition to transporting small molecule nutrients, MspA also increases uptake of the heavy metals Fe^3+^ and Cu^2+^ (44, 45). Excess Cu^2+^ and Zn^2+^ can be toxic to *M. tuberculosis* (45, 46). To test if metal toxicity contributes to reduced growth of the *eccD_5_* Tet-OFF pMV-*mspA* strain, we conducted growth curves in self-made 7H9 medium from which we omitted the Fe^3+^, Cu^2+^, and Zn^2+^ chemical components. We expect this medium to contain trace amounts of these metals (10, 47, 48). In 7H9 with trace metals and glycerol as the sole carbon source, the WT controls grew slowly and reached a lower cell density (Fig. 2B), but the *eccD_5_* Tet-OFF pMV-*mspA* strain grew similarly to WT and *eccD_5_* Tet-OFF pMV261 controls (Fig. 2D). Importantly, growth of the *eccD_5_* Tet-OFF pMV-*mspA* strain was not inhibited by Atc, even though EccD_5_ was depleted (Fig. 2D; Fig. S3B). In contrast, growth of the *eccD_5_* Tet-OFF pMV261 empty vector control was inhibited by Atc (Fig. 2D). Growth curves in 7H9 with trace metals and no added carbon showed that MspA does not enable use of other carbon sources in the 7H9 base medium (Fig. S3C and D). To examine which heavy metals inhibit growth of the *eccD_5_* Tet-OFF pMV-*mspA* strain, we conducted similar growth curves in self-made trace metals 7H9 medium, to which we added Fe^3+^, Cu^2+^, or Zn^2+^ at the same final concentration as in standard Middlebrook 7H9. Either Cu^2+^ or Zn^2+^, but not Fe^3+^, inhibited growth of *eccD_5_* Tet-OFF pMV-*mspA* relative to the WT pMV261 and *eccD_5_* Tet-OFF pMV261 controls (Fig. S4). Collectively, these data suggest that *M. tuberculosis* requires EccD_5_ for uptake of glycerol across the mycobacterial outer membrane. These data also suggest that *M. tuberculosis* requires ESX-5 to detoxify excess Cu^2+^ or Zn^2+^.

### *M. tuberculosis* requires ESX-5 for outer membrane localization of PPE51

**Fig 3:**
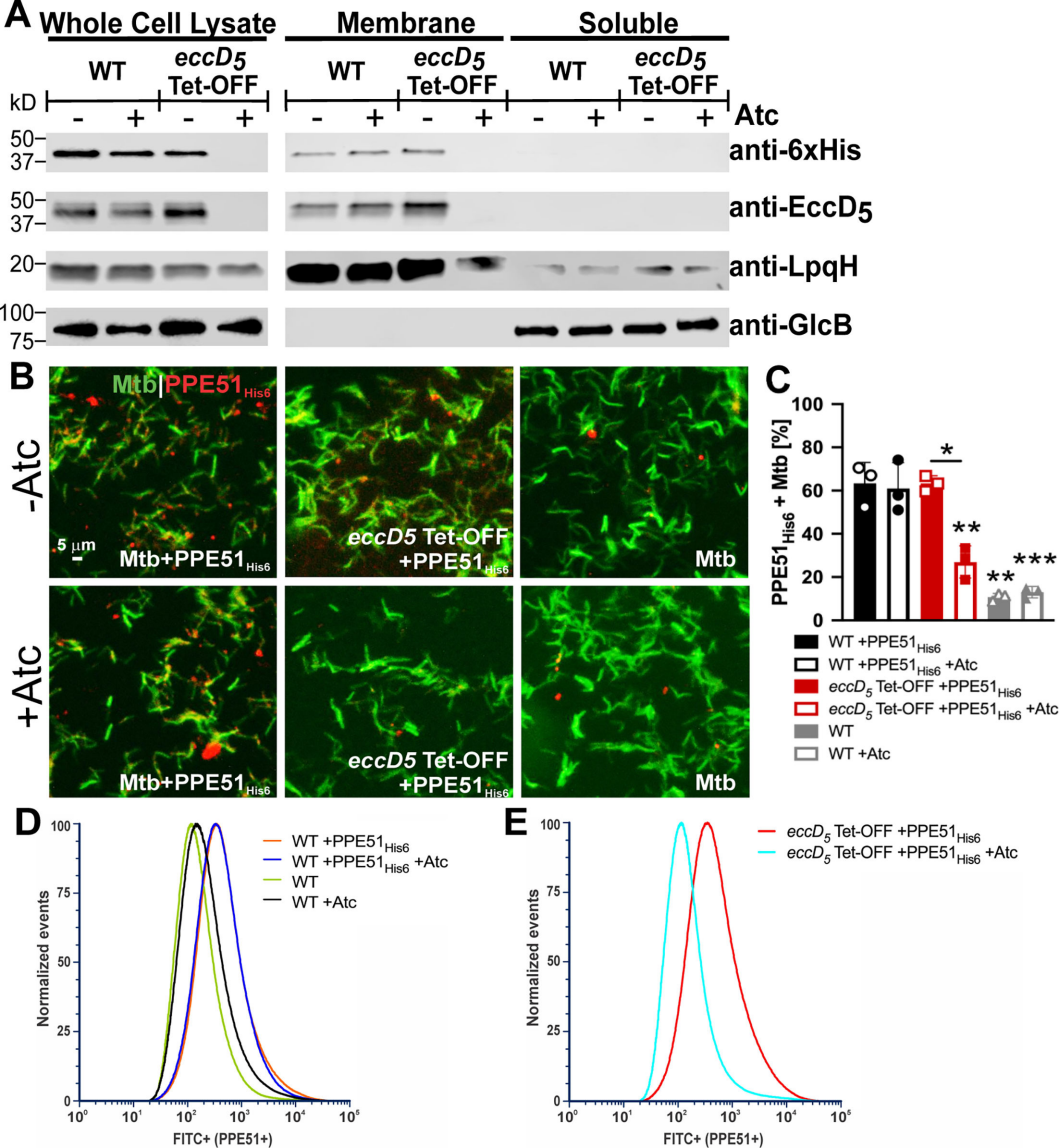
*M. tuberculosis* requires ESX-5 for outer membrane localization of PPE51. (**A**) Membrane localization of PPE51_His6_ by Western blotting. WT Erdman and *eccD_5_* Tet-OFF carrying pMV-*ppe51_His6_* were grown in Sauton’s medium ± 100 ng/mL anhydrotetracycline (Atc) to stationary phase for protein extraction. Whole cell lysates were separated into membrane and soluble fractions by ultracentrifugation. PPE51_His6_, EccD_5_, GlcB, and LpqH were detected in equal amounts of total lysate, membrane, and soluble fractions by Western blotting. GlcB and LpqH are soluble and membrane fraction loading controls, respectively. Results are representative of two independent experiments. (**B**) Detection of surface-accessible PPE51_His6_ in *M. tuberculosis* by fluorescence microscopy. The indicated *M. tuberculosis* strains were metabolically labeled with DMN-trehalose (green) and stained with monoclonal anti-6×His antibody to detect PPE51_His6_ (red). (**C**) Percentage of cells scored positive for PPE51_His6_ relative to the untagged WT strain quantified from the images in panel **B** (*n* = 3 technical replicates). Data are means ± standard deviations. Statistical analysis was performed between Atc (100 ng/mL) treated and untreated conditions for each strain (*P < 0.05, **P < 0.01; unpaired *t*-test). For each condition, statistical significance was calculated in comparison to WT *M. tuberculosis* with pMV-*ppe51_His6_* (**P < 0.01, ***P < 0.001; one-way ANOVA with Dunnett’s correction). (**D and E**) Surface-accessible PPE51_His6_ determined by flow cytometry using an anti-6×His antibody and Alexa Fluor 488 conjugated secondary antibody in *M. tuberculosis* cells grown ±100 ng/mL Atc. (**D**) Overlay of WT pMV-*ppe51_His6_* and untagged WT ± Atc. (**E**) Overlay of *eccD_5_* Tet-OFF pMV-*ppe51_His6_* ± Atc.

*M. tuberculosis* requires the PPE51 protein, which localizes to the cell surface, for uptake of and growth on glycerol and glucose (28). As we observed growth defects of EccD_5_-depleted *M. tuberculosis* on these carbon sources, we predicted that ESX-5 would export PPE51 to the outer membrane. To test this, we expressed PPE51 with a C-terminal 6×His tag (PPE51_His6_) in WT Erdman and *eccD_5_* Tet-OFF and performed Western blotting of soluble and membrane fractions. PPE51_His6_ was detected in the membrane fraction of WT *M. tuberculosis* and the *eccD_5_* Tet-OFF strain grown without Atc but was undetectable in the membrane fraction when EccD_5_ was depleted with Atc (Fig. 3A). PPE51_His6_ was also not detected in whole cell lysates upon EccD_5_ depletion (Fig. 3A), suggesting that PPE51_His6_ is not stable if it cannot be exported by ESX-5. The GlcB soluble and LpqH membrane fraction controls confirmed efficient fractionation and equivalent loading (Fig. 3A). These data suggest that *M. tuberculosis* requires ESX-5 for PPE51 export.

To confirm that ESX-5 exports PPE51 to the *M. tuberculosis* outer membrane, we conducted antibody surface staining followed by both microscopy and flow cytometry. PPE51_His6_ was readily detected on the surface of WT Erdman and on *eccD_5_* Tet-OFF grown without Atc, which was reduced by EccD_5_ depletion with Atc (Fig. 3B). Quantification revealed that Atc significantly reduced the percentage of *eccD_5_* Tet-OFF bacteria with detectable surface-localized PPE51_His6_ to the background level of WT *M. tuberculosis* lacking the PPE51_His6_ expression vector (Fig. 3C). Analysis of 50,000 individual cells by flow cytometry also showed that PPE51_His6_ was readily detected on the surface of WT *M. tuberculosis*, as indicated by an increased fluorescence intensity relative to bacteria lacking the pMV-*ppe51_His6_* expression vector (Fig. 3D). PPE51_His6_ was also detected on the surface of *eccD_5_* Tet-OFF grown without Atc but not when EccD_5_ was depleted with Atc (Fig. 3E). Collectively, these data indicate that *M. tuberculosis* exports PPE51 to the outer membrane via the ESX-5 system.

### *M. tuberculosis* requires ESX-5 to grow in and induce inflammatory cell death by macrophages

**Fig 4:**
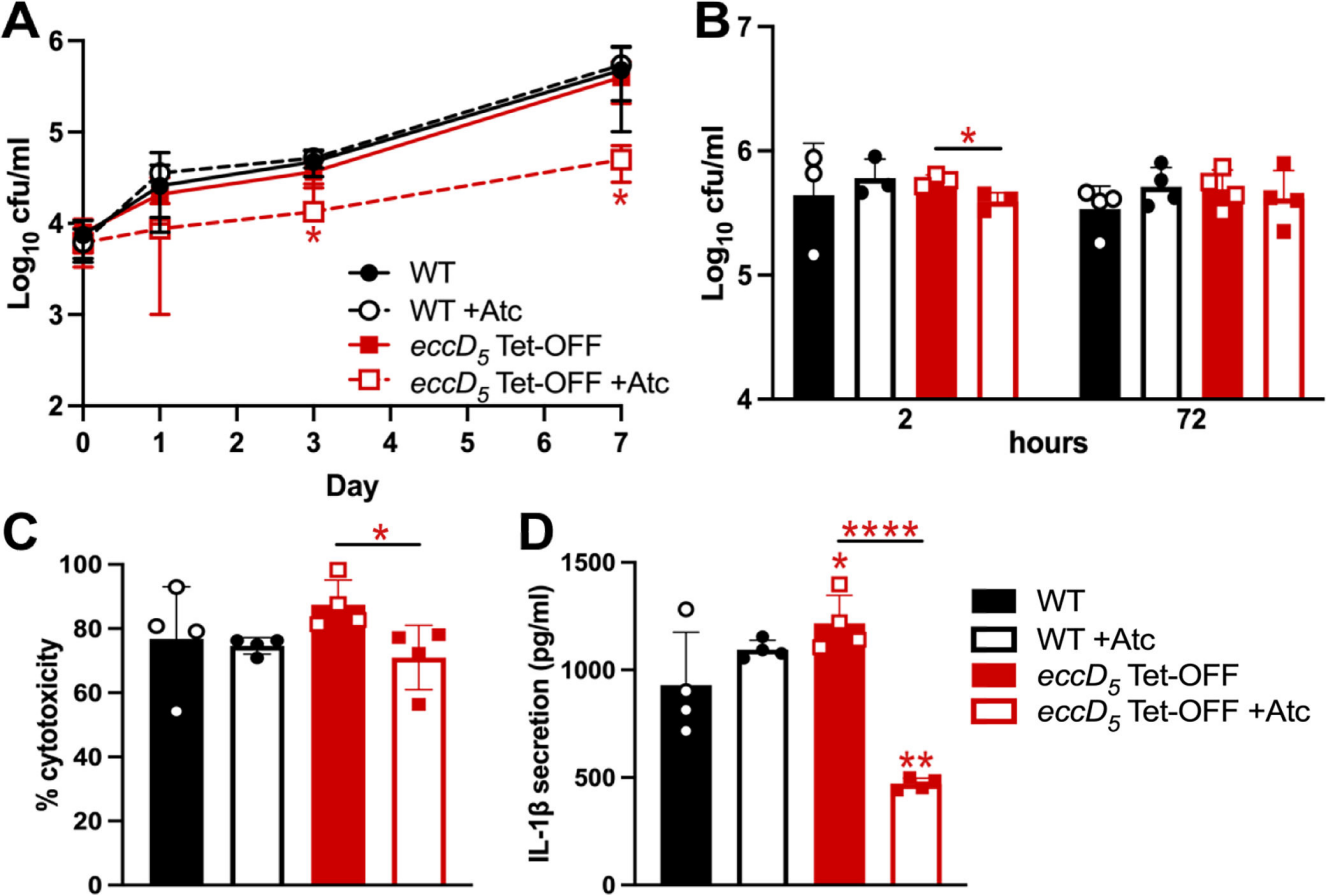
*M. tuberculosis* requires ESX-5 to grow in and induce inflammatory cell death of THP-1 cells. THP-1 cells were infected with the indicated *M. tuberculosis* strains grown in complete 7H9 ± 100 ng/mL anhydrotetracycline (Atc) and after infection were cultured ± 200 ng/mL Atc. (**A**) Cells were infected at a low MOI (1:20, bacteria:macrophage). Viable *M. tuberculosis* was determined at the indicated times post-infection by plating. Data are means ± standard deviations of three independent experiments. Statistical analysis was performed between Atc-treated and Atc-untreated conditions for each strain (* *P* < 0.05, unpaired *t*-test). (**B–D**). THP-1 cells were infected at an MOI of 1:1 (bacteria:macrophage). (**B**) Viable *M. tuberculosis* was determined by plating at 2 and 72 h. (**C**) Cytotoxicity was measured by a lactate dehydrogenase release assay. (**D**) Secreted IL-1β was measured by ELISA. Data are means ± standard deviations (*n* = 4). (**B–D**) Statistical analysis was performed between Atc-treated and Atc-untreated conditions (*P < 0.05, ****P < 0.0001; unpaired *t*-test) and to compare each strain and condition to the WT no-Atc control (*P < 0.05, **P < 0.001; one-way ANOVA with Dunnett’s correction). Complete results of statistical analyses are shown in Table S4.

To determine the importance of ESX-5 during *M. tuberculosis* growth within phagocytes, we used the human THP-1 monocyte cell line differentiated to macrophages. To assess *M. tuberculosis* replication, THP-1 cells were infected at a low MOI (1:20 bacteria:macrophage) with WT or *eccD_5_* Tet-OFF that was pre-grown ± Atc for 3 days to deplete EccD_5_. In our prior study, 3 days with Atc significantly repressed *eccD_5_* transcription (19). Depletion of EccD_5_ significantly reduced *M. tuberculosis* growth in resting THP-1 cells, with over a 1 log decrease in CFU at day 7 (Fig. 4A). *M. marinum* ESX-5 was implicated in the activation of inflammatory cell death and IL-1β cytokine release in THP-1 cells (13). To test if *M. tuberculosis* ESX-5 is similarly required to induce cell death, THP-1 cells were infected at a high MOI (1:1), and viable CFU, cytotoxicity, and IL-1β release were assessed. EccD_5_ depletion + Atc did not significantly alter *M. tuberculosis* survival at 72 h (Fig. 4B). However, EccD_5_ depletion significantly reduced THP-1 cell death induced by *M. tuberculosis* infection (Fig. 4C). In addition, THP-1 cells secreted significantly less IL-1β when infected with *M. tuberculosis* in which EccD_5_ was depleted (Fig. 4D). Collectively, these data demonstrate that ESX-5 plays critical roles in the replication of *M. tuberculosis* within resting phagocytes and in stimulating inflammatory responses by infected cells.

### *M. tuberculosis* requires ESX-5 to replicate, disseminate, and persist in aerosol-infected mice

**Fig 5:**
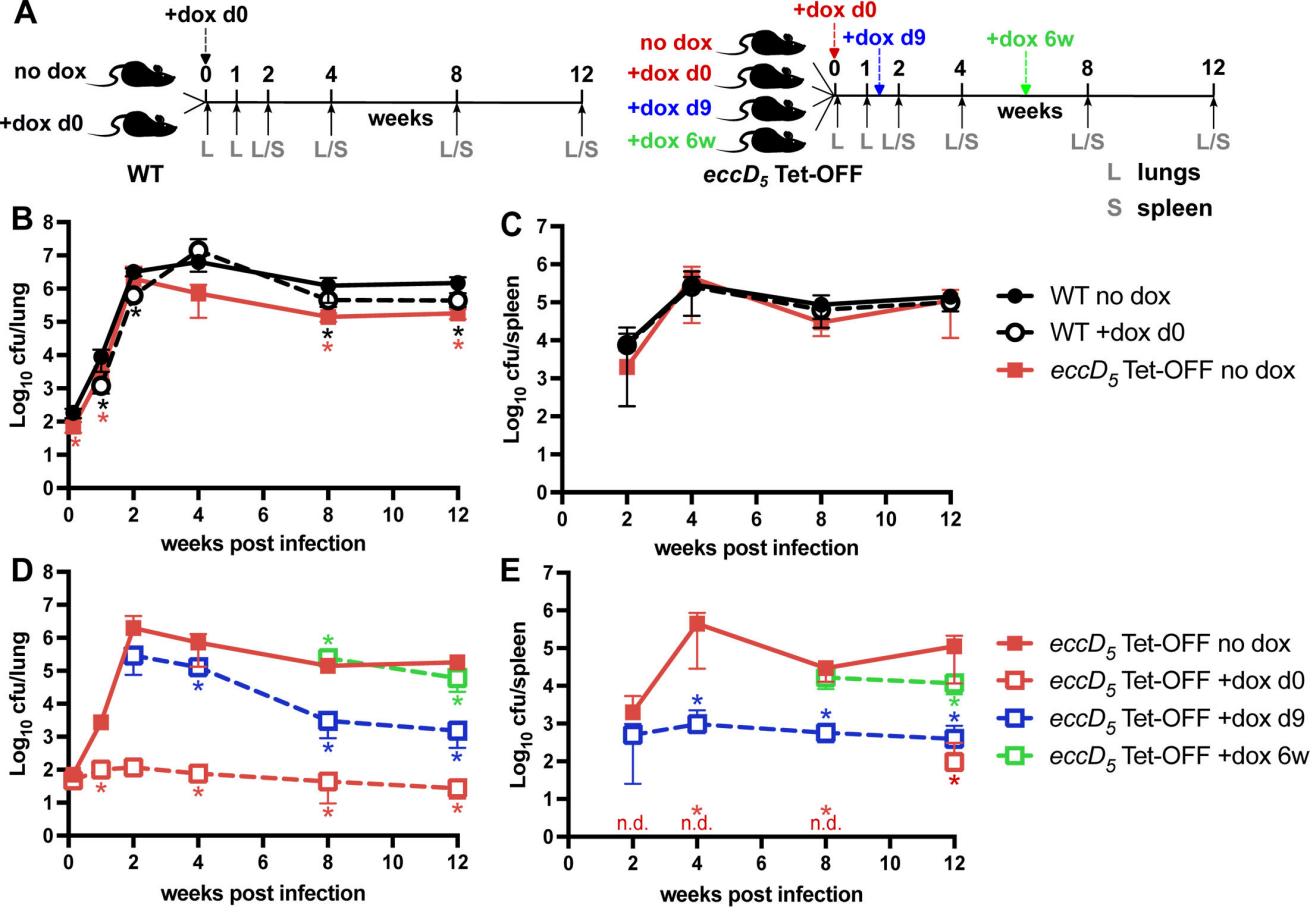
*M. tuberculosis* requires ESX-5 to replicate and disseminate in aerosol-infected mice. (**A**) Experiment design. Mice were infected with either WT Erdman or *eccD_5_* Tet-OFF by aerosol with ~100 CFU. Mice were divided into groups that were either untreated or treated with doxycycline (dox) starting at day 0, day 9, or week 6. Groups of mice (*n* = 6; 3 male, 3 female) were euthanized at the indicated times post-infection for collection of lung and spleen tissues. (**B–E**) Viable *M. tuberculosis* CFU determined by plating lung (**B and D**) or spleen (**C and E**) homogenates on 7H10 agar. Plates were incubated at 37°C for at least 4 weeks before counting CFU. Data are means ± standard deviations. Data for *eccD_5_* Tet-OFF no dox are reproduced in panels B–E to enable comparisons with both the WT controls and the + dox groups. Red n.d. in panel E indicates none detected for *eccD_5_* Tet-OFF + dox at day 0 (limit of detection = 3 CFU). (**B and C**) Statistical analysis was performed by a one-way ANOVA with Tukey’s correction at each time point (*P < 0.05). (**D and E**) One-way ANOVA with Dunnett’s correction was used to compare each dox-treated group to the *eccD_5_* Tet-OFF no-dox control (*P < 0.05). Complete results of statistical analyses are shown in Table S4.

To assess the role of ESX-5 in mammalian infection, we infected C57BL/6J mice by the aerosol route with either WT *M. tuberculosis* Erdman or *eccD_5_* Tet-OFF. To ensure infections were initiated by similar numbers of WT and *eccD_5_* Tet-OFF bacteria, we did not pre-deplete EccD_5_ with Atc prior to infecting the mice. To deplete EccD_5_ during infection, we used doxycycline (dox; 2,000 ppm in chow), a standard dose for conditional expression from Tet-regulated promoters in mice (49, 50) (Fig. 5A). For WT Erdman, dox treatment modestly impaired replication and persistence in the lungs (Fig. 5B) but did not significantly reduce dissemination to the spleen (Fig. 5C). Without dox treatment, the *eccD_5_* Tet-OFF strain replicated in the lungs similarly to the WT control during acute infection, achieving a similar lung burden at 2 weeks post-infection (Fig. 5B). The *eccD_5_* Tet-OFF strain also disseminated to the spleen similarly to the WT control (Fig. 5C). However, during chronic infection (8–12 weeks post-infection), significantly fewer *eccD_5_* Tet-OFF bacteria were recovered from the lungs of mice as compared to the WT no-dox control (Fig. 5B). These data suggest that *eccD_5_* expression from the Tet-OFF promoter is sufficient for normal ESX-5 function during acute infection. However, unregulated expression of *eccD_5_* from the Tet-OFF promoter may either increase or decrease ESX-5 activity during chronic infection, leading to modest attenuation.

 To determine at what stage(s) of infection *M. tuberculosis* requires ESX-5 activity, mice infected with the *eccD_5_* Tet-OFF strain were treated with dox starting at day 0 (initiation of infection), day 9 (acute infection), or week 6 (chronic infection) (Fig. 5A). We compared the number of viable *eccD_5_* Tet-OFF bacteria in dox-treated mice to the no-dox control. Treatment with dox starting at day 0 prevented replication of *eccD_5_* Tet-OFF bacteria in the lungs and dissemination to the spleens (Fig. 5D and E). The number of viable bacteria in the lungs increased slightly during the first 2 weeks (from 47 to 120 CFU, on average) but then slowly declined, reaching 28 CFU on average at 12 weeks post-infection (Fig. 5D). While *eccD_5_* Tet-OFF bacteria were not detected in the spleens of dox-treated mice through 8 weeks post-infection, at 12 weeks post-infection, we found viable CFU in the spleens of three out of six mice (Fig. 5E). We recovered only one colony from one mouse, which may represent cross-contamination, but two other mice had similar CFUs in the spleen and lungs, suggesting late dissemination of *eccD_5_* Tet-OFF bacteria (Fig. 5E). Both mice with high spleen CFU were male, but there was no obvious trend of increased CFU of the *eccD_5_* Tet-OFF strain in male mice in other mouse groups (Fig. S5).

To confirm that the *eccD_5_* Tet-OFF bacteria that disseminated to the spleen in dox-treated mice maintain Tet-repressible regulation of *eccD_5_*, we recovered colonies from the spleen of each mouse and tested *in vitro* growth and EccD_5_ protein production. Each *eccD_5_* Tet-OFF spleen isolate failed to grow on glycerol and glucose with Atc, similar to the parental control (Fig. S6A through F). Each *eccD_5_* Tet-OFF spleen isolate also exhibited EccD_5_ depletion upon treatment with Atc (Fig. S6G). In a Van10P vancomycin (Van) susceptibility assay for PDIM production (31), all isolates were equally resistant as the WT control (Fig. S6H), suggesting they produce PDIM.

To determine if these strains harbored secondary mutations that enabled dissemination to the spleen, we conducted whole genome sequencing using both Illumina short-read and Oxford Nanopore long-read technologies on one isolate from each mouse. In the isolate from mouse 2, which had 525 CFU in the spleen, we did not identify any secondary mutations. By short read sequencing, we identified a 21 bp deletion in *ppe3* in the isolate from mouse 3 (42 CFU) and short deletions in two genes encoding PE_PGRS proteins in the isolate from mouse 6 (1 CFU; *Erdman_1299* [*rv1157c*], 7 bp; *Erdman_1942*, 10 bp). However, long-read sequencing did not confirm these mutations, suggesting that they were errors due to the difficulty of mapping short sequencing reads to the repetitive *pe* and *ppe* genes. Because we did not identify secondary mutations in *eccD_5_* Tet-OFF strains recovered from dox-treated mouse spleens, it is possible that dissemination of ESX-5-deficient bacteria occurs randomly due to a loss of lung containment.

To determine the importance of ESX-5 during acute infection, we initiated dox treatment of mice infected with *eccD_5_* Tet-OFF at day 9 post-infection (Fig. 5A). We found that CFUs of the *eccD_5_* Tet-OFF strain were significantly reduced at 4–12 weeks compared to the no-dox control (Fig. 5D). Even in the 5 days between initiation of dox treatment and the 2 week time point, we observed reduced replication of *eccD_5_* Tet-OFF bacteria in the lungs (Fig. 5D) and reduced dissemination to the spleen (Fig. 5E). After the 2 week time point, dox treatment caused clearance of *eccD_5_* Tet-OFF bacteria from the lungs (Fig. 5D). However, we did not observe any reduction in *eccD_5_* Tet-OFF bacteria in the spleens over time (Fig. 5E). At the dose we used, dox accumulates to a sufficient concentration in the spleen to fully repress transcription from Tet-repressible promoters (50). These data suggest that *M. tuberculosis* requires ESX-5 activity for replication and survival within the lung during acute phase replication, but that ESX-5 may be dispensable for *M. tuberculosis* survival in the spleen.

To determine the role of ESX-5 during chronic infection, mice infected with *eccD_5_* Tet-OFF were treated with dox starting at 6 weeks post-infection (Fig. 5A). We observed modest but statistically significant reductions in viable *eccD_5_* Tet-OFF bacteria recovered from the lungs and spleens of dox-treated mice at 12 weeks post-infection relative to the no-dox controls (Fig. 5D and E). These data suggest that *M. tuberculosis* may have a reduced requirement for ESX-5 during chronic infection. Alternatively, *M. tuberculosis* may not need to produce new ESX-5 membrane complexes during chronic infection because it is in a slow-growing state (51), resulting in relatively limited impact of *eccD_5_* transcriptional repression on chronic phase persistence.

## DISCUSSION  

*M. tuberculosis* requires ESX-5 for growth in standard culture conditions *in vitro*, which has complicated analysis of its roles during infection. Here, using conditional expression of the EccD_5_ membrane component, we show that ESX-5 has broad functions in *M. tuberculosis* physiology and pathogenesis. We demonstrate that *M. tuberculosis* requires ESX-5 to grow on multiple carbon sources *in vitro*, including glycerol and glucose that are the carbon sources in standard Middlebrook media, which may explain its essentiality. We show that PPE51 is a substrate of ESX-5 that associates with the *M. tuberculosis* outer membrane, confirming a recent report that *M. marinum* ESX-5 exports PPE51 (52). Failure to export PPE51, which is required for glucose and glycerol uptake (28), likely explains why EccD_5_-depleted *M. tuberculosis* cannot grow on these carbon sources. As in *M. marinum*, expression of the outer membrane porin MspA rescued growth of EccD_5_-depleted *M. tuberculosis* on glycerol, suggesting that ESX-5 functions to export nutrient transporters to the outer membrane. We show that EccD_5_ depletion prevents *M. tuberculosis* replication in resting macrophages and in the lungs of mice. Importantly, depletion of EccD_5_ causes *M. tuberculosis* clearance from mouse lungs, demonstrating that ESX-5 activity is critical for pathogenesis. Determining whether ESX-5 is important for nutrient acquisition or exporting or secreting “effector” proteins that directly manipulate host cell functions during infection will require identifying and characterizing additional ESX-5 substrates.

Our data support a role for *M. tuberculosis* ESX-5 in activating macrophage inflammatory responses, including production of IL-1β, which was previously described in *M. marinum* (13). However, our data identified key differences from the *M. marinum* model that highlight the importance of characterizing ESX-5 function in *M. tuberculosis*. An ESX-5-deficient *M. marinum* strain was hypervirulent in adult zebrafish (53), which contrasts with strongly reduced virulence of EccD_5_-deficient *M. tuberculosis* in mice. Our EccD_5_-deficient *M. tuberculosis* lacks a central ESX-5 membrane component, while the *M. marinum* mutant lacked the cytosolic ESX-5 chaperone EspG_5_ (53). The *M. marinum* mutant may retain a functional ESX-5 membrane complex with at least partial ESX-5 secretion activity. Alternatively, because ESX-5 is also essential in *M. marinum* (20), the *M. marinum espG_5_* mutant could harbor secondary mutations that enable growth *in vitro* and increase virulence in zebrafish.

Our data also show that the *M. smegmatis* porin MspA cannot fully rescue growth of ESX-5-deficient *M. tuberculosis* in 7H9 medium with glycerol as the carbon source, which contrasts with *M. marinum* in which expression of MspA enabled deletion of either *eccC_5_* or *mycP_5_* (20). These data suggest either that ESX-5 has distinct functions in *M. tuberculosis* and *M. marinum* or that *M. marinum* strains expressing MspA harbor compensatory secondary mutations. Indeed, we observed an extended lag phase for our *eccD_5_* Tet-OFF pMV-*mspA* strain grown in 7H9 with glycerol, during which it may have acquired secondary mutations or undergone transcriptional adaptation.

MspA fully rescued growth of EccD_5_-deficient *M. tuberculosis* in medium with trace heavy metals, indicating that ESX-5 is required for metal resistance. Expression of MspA sensitized ESX-5-deficient *M. tuberculosis* to Cu^2+^ and Zn^2+^, but not Fe^3+^. Cu^2+^ and Zn^2+^ are concentrated in macrophage phagosomes and restrict *M. tuberculosis* replication (46, 47, 54). While ATPases that pump Cu^2+^ and Zn^2+^ across the *M. tuberculosis* inner membrane have been identified (46, 55, 56), mechanisms of Cu^2+^ and Zn^2+^ efflux across the outer membrane are unknown. Metal efflux may be facilitated by PE and/or PPE protein substrates of the ESX-5 system. The *eccD_5_* Tet-OFF strain also had a modest growth defect in standard 7H9 medium with glycerol as the carbon source even without Atc, which was not evident for the *eccD_5_* Tet-OFF pMV261 empty vector control. We used kanamycin (Kan) to maintain pMV261, which chelates Cu^2+^ (57). The *eccD_5_* Tet-OFF strain also grew similarly to the WT control on glycerol in minimal salt medium, which has a 10-fold lower concentration of Zn^2+^ than 7H9 and no added Cu^2+^. These data suggest that even partial impairment of ESX-5 secretion can sensitize *M. tuberculosis* to metal stress.

 While *M. tuberculosis* requires ESX-5 to export PPE51, the phenotypes associated with EccD_5_ depletion in macrophages and mice are unlikely to be due exclusively to reduced PPE51 export. PPE51 was implicated in inhibiting macrophage autophagy, but an *M. tuberculosis ∆ppe51* mutant induced higher IL-1β release from infected mouse macrophages and was only modestly attenuated during chronic infection in intranasally infected mice (58). *M. tuberculosis* also does not require catabolism of glycerol or glucose to replicate in mouse lungs during acute infection, and mutants that cannot use glucose are only modestly attenuated during chronic infection (59, 60). These data suggest that other ESX-5 substrates, besides PPE51, support *M. tuberculosis* replication and persistence in mice.

ESX-5 may be required during acute infection to enable use of fatty acids that are considered primary carbon sources in infected cells (61). EccD_5_-depleted *M. tuberculosis* likely fails to grow on the model fatty acid ester Tween-40 due to reduced export of lipase or esterase enzymes that hydrolyze Tween-40 to release free fatty acids that are taken up by Mce1 (62). Many PE and PPE proteins have lipase or esterase activity (63–65) and are likely to be exported to the outer membrane by ESX-5. Several PE and PPE proteins have also been implicated in the acquisition of heme iron (37, 38). Our future studies will include identification of additional ESX-5 substrates that are required to use other nutrients, including carbon sources and heme, and determination of the importance of ESX-5-dependent export of these substrates during infection.

 Our data show that *M. tuberculosis* requires ESX-5 for replication in the lungs and dissemination to the spleen in acutely infected mice, suggesting that ESX-5 is critical for host-pathogen interactions from the earliest stage of infection. The *M. tuberculosis* ESX-3 secretion system is similarly required during acute infection, as an ∆*esx-3* mutant was rapidly cleared from the lungs and failed to disseminate to the spleen in aerosol-infected C57BL/6 mice (9). *M. tuberculosis* ESX-3, like ESX-5, is essential for growth in standard culture conditions *in vitro* due to its role in iron acquisition (9). In contrast, *M. tuberculosis* mutants lacking ESX-1, which is considered a primary virulence factor, replicate in the lungs and spleens of C57BL/6 mice but at a slower rate than WT bacteria (66–68). Our data suggest that ESX-5 is at least as important in *M. tuberculosis* pathogenesis as these other ESX secretion systems and support further characterization of its functions during infection.

 We were surprised that EccD_5_ depletion at the initiation of infection did not cause clearance of *M. tuberculosis* from mouse lungs. We did not deplete EccD_5_ before infecting the mice, so the bacteria may remain viable due to pre-existing ESX-5 secretion complexes, which are not disrupted by dox treatment that only represses *eccD_5_* transcription. At this early stage of infection, *eccD_5_* transcriptional repression may restrict *M. tuberculosis* within resting alveolar macrophages. EccD_5_ depletion limited production of the inflammatory cytokine IL-1β by infected THP-1 cells. Infected alveolar macrophages must produce IL-1β to enable their migration from the airways to the lung interstitium (69). We speculate that because ESX-5-deficient *M. tuberculosis* fails to induce IL-1β, infected alveolar macrophages do not migrate into the lung tissue and therefore fail to activate an adaptive immune response. We observed dissemination to the spleen in a subset of animals at a late time point in these mice, which we could not attribute to any secondary mutations. Alveolar macrophages are very long-lived in mice, with a life span of at least several months (70). When these infected cells die, *M. tuberculosis* may be taken up by other cell types that can escape from the lung to enable dissemination to the spleen.

Importantly, we observed clearance of EccD_5_-depleted *M. tuberculosis* from the lungs of mice that were treated with dox during acute infection. ESX-5 may be particularly important for *M. tuberculosis* to resist stressors in activated macrophages since we observed clearance after 2 weeks post-infection, which coincides with recruitment of T cells producing IFN-γ to the lungs (71). However, *eccD_5_* Tet-OFF bacteria persisted in the spleen during dox treatment, even though the dox concentration is high enough to repress the Tet-OFF promoter (50). *M. tuberculosis* may infect a different cell type in the spleen, in which it does not require ESX-5 activity, or may enter a slowly replicating state, in which it can persist using pre-existing ESX-5 secretion complexes. This may also explain why *eccD_5_* transcriptional repression during chronic infection caused only modest reductions in bacterial burdens, as *M. tuberculosis* replicates slowly in this phase of infection (51). Alternatively, ESX-5 activity could be less important during chronic infection if it primarily enables *M. tuberculosis* growth by exporting outer membrane transporters or enzymes for nutrient acquisition. Distinguishing between these possibilities will require either using dual control systems for conditional protein degradation of ESX-5 components, which may be possible for the cytosolic EspG_5_ chaperone and EccA_5_ ATPase, or developing small molecule inhibitors of ESX-5.

Finally, our data indicate that ESX-5 is a strong candidate for development of new antitubercular drugs. Small molecule screens identified inhibitors of *M. tuberculosis* ESX-1 which act as antivirulence drugs and limit bacterial growth in cell culture infection models (72–74). Inhibitors of ESX-5 might be more effective, as loss of ESX-5 function prevents *M. tuberculosis* growth and leads to clearance from infected mice. A screen for small molecules that inhibit *M. marinum* ESX-5 was reported, but the compounds identified caused dysregulated export of the LipY lipase reporter and did not specifically inhibit ESX-5 (75). Similar screens to those reported for ESX-1 could be done to identify small molecule inhibitors of *M. tuberculosis* ESX-5. Structures of the ESX-5 inner membrane complex exist to guide drug development (14, 15, 76), but the mechanisms by which ESX-5 substrates become incorporated into or pass through the *M. tuberculosis* outer membrane remain unknown. Identifying the ESX-5 outer membrane components will be important, as small molecules targeting these components would not need to pass through the outer membrane permeability barrier to have activity. Our future studies will seek to define these ESX-5 outer membrane components and determine how they mediate ESX-5 substrate export.

## MATERIALS AND METHODS

### Bacterial strains and culture conditions

WT *M. tuberculosis* Erdman, *eccD_5_* Tet-OFF (19), and derivative strains (Table S1) were grown at 37°C with aeration in Middlebrook 7H9 medium (BD Difco) supplemented with 10% albumin-dextrose-saline, 0.5% glycerol, and 0.1% Tween-80 (complete 7H9) or on Middlebrook 7H10 agar (BD Difco) supplemented with 10% oleic acid-albumin-dextrose-catalase (OADC, BD Biosciences) and 0.5% glycerol, unless otherwise noted. Frozen stocks were prepared by growing cultures to late-exponential phase and adding glycerol to 15% final concentration and were stored at −80°C. Antimicrobials were used at the following concentrations: Kan 25 μg/mL for agar or 15 μg/mL for liquid, hygromycin (Hyg) 50 μg/mL, and cycloheximide 100 μg/mL.

### Growth curves

Bacteria were grown to mid-exponential phase (OD_600_ of ~0.5) in complete 7H9 medium, then diluted to OD_600_ = 0.05 in fresh complete 7H9 medium ± 100 ng/mL Atc (Sigma) and grown for 3 days to pre-deplete EccD_5_. Bacteria were collected by centrifugation (10 min, 2,850 × *g*), washed twice in PBS with 0.01% tyloxapol (Sigma), then inoculated in triplicate at OD_600_ = 0.01 in Middlebrook 7H9 base (BD Difco) containing 0.01% tyloxapol and a sole carbon source: 0.5% glycerol, 0.2% glucose, 0.2% Tween-40 (Sigma), or 0.01% cholesterol (Sigma). Cholesterol stocks were prepared at 100 mg/mL in a 1:1 EtOH:tyloxapol solution at 80°C as described (77). Fresh cholesterol was added to 0.01% final concentration every 2–3 days from a cholesterol stock pre-warmed to 80°C. To test growth in minimal salt medium, bacteria grown in complete 7H9 medium ± 100 ng/mL Atc to pre-deplete EccD_5_ were washed twice in minimal medium without a carbon source [1 g/L KH_2_PO_4_, 2.5 g/L Na_2_HPO_4_, 0.5 g/L (NH_4_)_2_SO_4_, 0.15 g/L asparagine, 50 mg/L ferric ammonium citrate, 0.5 g/L MgSO_4_ •7H_2_O, 0.5 mg/L CaCl_2_, and 0.1 mg/L ZnSO_4_ •7H_2_O, 0.05% tyloxapol], then diluted in duplicate at OD_600_ = 0.05 in minimal medium with no added carbon, 0.2% glycerol, or 0.2% glucose ± 100 ng/mL Atc. To test heavy metal toxicity, bacteria were grown in self-made trace metals 7H9 [2.5 g/L Na_2_HPO_4_, 1.0 g/L KH_2_PO_4_, 0.5 g/L monosodium glutamate, 0.5 g/L (NH_4_)_2_SO_4_, 0.05 g/L MgSO_4_ •7H_2_O, 1.0 mg/L pyridoxine HCl, 0.5 mg/L biotin, and 0.5 mg/L CaCl_2_], made by omitting the Fe^3+^, Cu^2+^, and Zn^2+^ components of standard 7H9 medium. To test the toxicity of individual heavy metals, Fe^3+^ (0.04 g/L ferric ammonium citrate), Cu^2+^ (1 mg/L CuSO_4_ •5H_2_O), or Zn^2+^ (1 mg/L ZnSO_4_ •7H_2_O) were added to the self-made trace metal 7H9 at the same concentration as in standard Middlebrook 7H9. Fresh Atc (100 ng/mL) was added to cultures every 7 days to maintain *eccD_5_* transcriptional repression. The OD_600_ of cultures was measured every 2–3 days. For minimal medium, cultures were serially diluted and plated on 7H10 agar at days 0, 7, and 14. Plates were incubated at 37˚C for 3–4 weeks before counting CFU.

### Cloning and strain construction

Plasmids used in this study are listed in Table S2. The pMV-*mspA* vector was generated by cloning *M. smegmatis mspA* and the mycobacterial optimal promoter *P_smyc_* from pMN016 (27) into the episomal vector pMV261. The pMV261 vector was digested with XbaI and ClaI to remove the P*_hsp60_* promoter. *M. smegmatis mspA* and *P_smyc_* were removed from pMN016 by digestion with XbaI and ClaI. The digested products were gel purified (QIAquick Gel Extraction, Qiagen), ligated with T4 DNA ligase (NEB), and transformed in *Escherichia coli* DH5α. The pMV-*mspA* vector was confirmed by XbaI/ClaI digest and Sanger sequencing.

The pMV-*ppe51_His6_* vector was made by cloning *ppe51_His6_* from the integrating vector pMV306-NHK-*ppe51_His6_* (28) into pMV261. Plasmids were digested with XbaI and HindIII, the pMV261 vector and *ppe51_His6_* insert were gel purified (QIAquick gel extraction; Qiagen), ligated with T4 DNA ligase, and transformed in *E. coli* DH5α. The pMV-*ppe51_His6_* vector was confirmed by XbaI/HindIII digest and Sanger sequencing.

The pMV261, pMV-*mspA*, and pMV-*ppe51_His6_* plasmids were transformed into WT Erdman and *eccD_5_* Tet-OFF by electroporation as described (78). Transformants were selected by plating on 7H10 with Kan (WT) or 7H10 with Kan and Hyg (*eccD_5_* Tet-OFF) and confirmed by PCR using primers listed in Table S3.

### Protein extraction, fractionation, and Western blotting

*M. tuberculosis* strains were grown in complete 7H9 medium to mid-exponential phase, washed twice in Sauton’s medium (4 g/L L-asparagine, 0.5 g/L potassium phosphate monobasic, 0.5 g/L MgSO_4_ •7H_2_O, 2 g/L citric acid, 0.05 g/L ferric ammonium citrate, 60 mL/L glycerol, and 1 mg/L ZnSO_4_) with 0.1% Tween-80 (Tw-80) and resuspended at OD_600_ = 0.2 in 30 mL Sauton’s medium + 0.1% Tw-80 ± 100 ng/mL Atc. Cultures were grown at 37°C to late-logarithmic phase (OD_600_ = ~1.2). Bacteria were pelleted (2,850 × *g*, 10 min), resuspended in 30 mL Sauton’s medium without detergent, then grown with aeration for 6 days at 37°C. Bacteria were collected by centrifugation (4,100 × *g*, 15 min, 4°C) and washed twice in ice-cold PBS. Cell pellets were resuspended in 1 mL ice-cold PBS containing Complete EDTA-Free Protease Inhibitors (Roche), and bacteria were lysed by bead beating. Large cell debris was removed by centrifugation, and lysates were filter sterilized with a 0.2 μm Nanosep MF Centrifugal Filter (Pall Life Sciences) as described (79). Whole cell lysates were separated into membrane and soluble fractions by two rounds of ultracentrifugation (100,000 × *g*, 1 h, 4°C) as described (37). The final membrane pellet was resuspended in PBS with protease inhibitors and 1% SDS.  

Protein concentrations in whole cell lysates and soluble fractions were determined by BCA assay (Pierce). Equal amounts of total protein or equivalent volumes of the membrane fraction based on whole cell lysate protein concentrations were separated by SDS-PAGE on AnyKD gels (Bio-Rad) and transferred to 0.2 μm nitrocellulose membranes. Membranes were blocked in PBS with 0.1% Tween-20 (PBST-20) with 5% non-fat milk powder for 1 h, washed with PBST-20, and probed overnight at 4°C with primary antisera diluted in PBST-20 with 2.5% non-fat milk powder. Primary antisera were used at the following dilutions: rabbit anti-6×His 1:5,000 (ZooMab ZRB2297, Sigma-Aldrich), rabbit anti-EccD_5_ 1:1,000 (19), mouse anti-LpqH 1:1,000 (NR-13792, clone IT-54; BEI Resources), mouse anti-GlcB 1:500 (NR-13799, BEI Resources), or mouse anti-GroEL2 1:1,000 (NR-13657, clone IT-70; BEI Resources). Membranes were washed with PBST-20, then probed for 1 h with horseradish peroxidase-conjugated secondary antibody (goat antirabbit IgG 31466 or goat antimouse IgG 31431, Thermo Fisher) at 1:10,000 dilution in PBST-20 with 2.5% non-fat milk powder. Membranes were washed with PBST-20, incubated for 5 min with SuperSignal Pico West chemiluminescent substrate (Thermo Fisher), and immediately imaged using an Odyssey Fc Imaging System (LI-COR) with LI-COR Image Studio software.

### Detection of surface-accessible PPE51_His6_ in *M. tuberculosis* by flow cytometry

Surface detection of PPE51_His6_ in *M. tuberculosis* Erdman and derivative strains was performed as previously described (3). Bacteria were grown in Middlebrook 7H9 supplemented with 10% OADC, 0.5% glycerol, and 0.02% tyloxapol. To maintain PDIM production, 0.1 mM sodium propionate was added to the medium (31). Once the cultures reached an OD_600_ of ~0.8, bacteria were pelleted and washed once in Sauton’s medium containing 0.02% tyloxapol. The cells were then cultured in the same medium with the required antibiotics until an OD_600_ of ~1.0 was obtained. Cultures were passed through a 5 μm syringe filter (Millipore) and allowed to grow until an OD_600_ of ~1.0 was obtained. Cells were fixed with 4% paraformaldehyde for 1 h and blocked in 2.5% normal goat serum for 30 min. Subsequently, they were incubated with anti-His Tag antibody (1:50, ABclonal #AE003) for 1 h followed by incubation with Alexa Fluor 488-conjugated goat antimouse IgG (H + L) secondary antibody (1:500, Invitrogen) for another hour. Between each step, bacteria were washed three times with 1× PBS. For each sample, an unstained control (autofluorescence) was included, and 50,000 events were acquired per sample using a flow cytometer (LSRFortessa, BD Biosciences). Data were exported as FCS files and analyzed with FCS Express 7 software, and surface-accessible PPE51_His6_ was displayed as histograms.

### Analysis of surface-accessible PPE51_His6_ in *M. tuberculosis* by fluorescence microscopy

*M. tuberculosis* strains were cultured as for flow cytometry experiments. After passage through the 5 μm syringe filter (Millipore), bacteria were grown in Sauton’s medium containing 0.02% tyloxapol until an OD_600_ of ~0.8 was obtained. Cells were then stained with 100 μg/mL DMN-trehalose (80) and incubated overnight at 37°C. Following staining, cells were fixed with 4% paraformaldehyde for 1 h, blocked with 2.5% normal goat serum for 30 min, and sequentially incubated with anti-His Tag antibody (1:50, ABclonal #AE003) and Alexa Fluor 594-conjugated goat antimouse IgG (1:300, Invitrogen), each for 1 h. Bacteria were washed three times with PBS between each step. After staining, bacteria were smeared on coverslips and mounted on glass slides using ProLong Glass Antifade Mounting (Invitrogen). The samples were then analyzed using quantitative fluorescence microscopy (BioTek Cytation 5 Cell Imaging Multimode Reader with ×60 objective). Image analysis was done with Gen5 software. A primary mask was applied based on GFP fluorescence (representing *M. tuberculosis*) with object size thresholds set between 2 and 10 μm. PPE51_His6_ expression was visualized using a TRITC filter, and this fluorescence was used as the secondary mask. Mean TRITC intensity was measured within the primary mask. Scatter plots were generated in Gen5, and subpopulations with elevated fluorescence were gated relative to untagged WT *M. tuberculosis*. Bar graphs were plotted from the data obtained from Cytation 5 analysis using GraphPad Prism.

### Growth in THP-1 macrophages

The THP-1 (ATCC TIB-202) human monocytic cell line was cultured in RPMI base (ATCC or Gibco) supplemented with non-heat-treated 10% fetal bovine serum (Corning or Gibco) (hereafter referred to as RPMI) in a humidified incubator at 37°C with 5% CO_2_. THP-1 cells were differentiated into monocyte-derived macrophages with 50 nM phorbol 12-myristate-13-acetate (PMA, Sigma) for 24 h in 96-well tissue culture plates for experiments at 1:20 MOI or 24-well tissue culture plates for experiments at 1:1 MOI. Differentiated cells were washed twice with Hank’s Balanced Salt Solution (Corning), given fresh RPMI, and rested for 72 h before infection.

Bacteria were grown from frozen stocks in complete 7H9 to mid-exponential phase (OD_600_ = 0.4–0.7), then diluted to OD_600_ = 0.05 in complete 7H9 ± 100 ng/mL Atc and grown for 72 h prior to the infection. Bacteria were pelleted by centrifugation (2,850 × *g*, 10 min), resuspended in phosphate-buffered saline with 0.05% Tween-80 (PBS-T), declumped by centrifugation (58 × *g*, 5 min), and diluted in RPMI at the appropriate density for infection. The THP-1 cell culture media were refreshed with RPMI ± 200 ng/mL Atc. Infections were initiated at an MOI = 1:20 (bacteria:macrophage) or MOI = 1 for 2 h at 37°C with 5% CO_2_. Extracellular bacteria were removed by three washes with pre-warmed Dulbecco’s PBS (Gibco); RPMI ± 200 ng/mL Atc was replaced; and cells were incubated at 37°C with 5% CO_2_. For experiments at an MOI = 1:20, viable intracellular bacteria were enumerated at 2 h, 1, 3, and 7 days post-infection. For experiments at an MOI = 1, bacteria were enumerated at 2 and 72 h post-infection. THP-1 cells were lysed at 37°C for 10 min with 0.1% Tween-80. Monolayers were visually inspected for lysis before serially diluting the lysate in PBS-T and plating on 7H10 to recover viable bacteria. Plates were incubated for at least 3 weeks at 37°C before counting the CFU.

### Cell death and cytokine release assays

PMA-differentiated THP-1 cells infected at an MOI of 1 were assessed for cellular necrosis by measuring lactate dehydrogenase (LDH) release and for production of the inflammatory cytokine IL-1β at 3 days post-infection. Cell culture supernatants from infected cells and uninfected controls were filter sterilized with a 0.2 μm Nanosep MF centrifugal filter (14,000 × *g*, 3 min, 4°C; Pall Life Sciences). Supernatants were aliquoted and frozen at −80°C until use. LDH was measured with a CytoTox 96 Non-Radioactive Cytotoxicity Assay kit (Promega). Uninfected THP-1 control cells were lysed with 1× lysis solution provided with the kit for 45 min at 37°C to determine the maximal LDH release. Percent cytotoxicity was calculated as [LDH in culture supernatants (OD_492_) / maximal LDH from lysed uninfected cells (OD492)] × 100. Human IL-1β was quantified in culture supernatants by ELISA (Invitrogen).

### PDIM assays

To directly measure PDIM production, *M. tuberculosis* cultures grown in 10 mL complete 7H9 medium to mid-logarithmic phase were labeled for 48 h with 10 μCi of [1-^14^C] propionic acid, sodium salt (specific activity 50–60 mCi/mmol; American Radiolabeled Chemicals, Inc.) prior to extraction of apolar lipids as described (25). Labeled lipids were separated by thin-layer chromatography and detected by phosphor imaging as described (81). A Van10P assay was also used to indirectly assess PDIM production based on Van susceptibility in the presence of propionate (31). Bacteria grown to late-exponential phase (OD_600_ ~0.8) in complete Middlebrook 7H9 were diluted 1:100 in 7H9 containing 0.1 mM propionate ± 10 μg/mL Van. The OD_600_ was measured at 14 days, and Van10P % growth was calculated as (Van10 − *P* OD_600_ / Van0 − *P* OD_600_) × 100.

### Mouse infections

Male and female C57BL/6J mice 6 weeks of age were purchased from the Jackson Laboratory, USA, and infected by the aerosol route with ~100 CFU of *M. tuberculosis* Erdman or *eccD_5_* Tet-OFF using an Inhalation Exposure System (GlasCol) as described (82). Bacteria grown to mid-exponential phase (OD_600_ of ~0.5) were washed once with PBS-T and diluted to OD_600_ = 0.007 in PBS-T for aerosol infection. Groups of mice were treated with dox chow (irradiated Teklad Global 2018 rodent diet containing 2,000 ppm dox; Envigo) starting at day 0 (immediately after infection), day 9, or day 42 (week 6). Dox chow was stored at 4°C until use and was replaced weekly. Groups of mice (*n* = 6; 3 male, 3 female) were euthanized by CO_2_ overdose at 24 h and 1, 2, 4, 8, and 12 weeks post-infection to recover lung and spleen tissues. Organs were homogenized in PBS-T, and bacterial CFU were determined by plating serially diluted homogenates on 7H10 containing cycloheximide. Colonies were counted after at least 4 weeks of incubation at 37°C.

### Whole genome sequencing

Genomic DNA was extracted from *M. tuberculosis eccD_5_* Tet-OFF and single *eccD_5_* Tet-OFF isolates from the spleens of mice treated with dox starting at day 0. Strains were grown to late-logarithmic phase (OD_600_ = 1.0), and DNA was extracted by the CTAB-lysozyme method (83). DNA was cleaned with the Genomic DNA Clean and Concentrator Kit 25 (Zymo) and submitted to Microbial Genome Sequencing Center (MiGS, now SeqCenter, Pittsburgh, PA, USA; *eccD_5_* Tet-OFF) or SeqCoast Genomics (Portsmouth, NH, USA; *eccD_5_* Tet-OFF spleen isolates) for library preparation and Illumina sequencing. MiGS conducted library preparation with the Illumina DNA Prep kit and IDT 10 bp UDI indices and sequencing on an Illumina NextSeq 2000 (2 × 151 bp reads). Demultiplexing, quality control, and adapter trimming were conducted using bcl-convert (v.3.9.3). SeqCoast conducted library preparation with the Illumina DNA Prep tagmentation kit and unique dual indices and sequencing on the Illumina NextSeq 2000 using a 300 cycle flow cell kit (2 × 150 bp reads) with 1%–2% PhiX control spiked in for optimal base calling. Read demultiplexing, trimming, and run analytics were performed using DRAGEN (v.3.10.12). To generate a consensus sequence for each strain, paired reads were mapped to the *M. tuberculosis* Erdman reference genome (NC_020559.1) using the “map to reference” function in Geneious Prime 2021 software (Biomatters, Ltd.) as previously described (81). To identify single-nucleotide polymorphisms or small insertions and deletions, whole genome alignments were performed with the Erdman reference genome, the published Tischler WT Erdman genome (81), and the *eccD_5_* Tet-OFF strains using the “Align Whole Genomes” function with the default Mauve genome parameters as described (81).

For Oxford Nanopore Technology (ONT) long-read sequencing, genomic DNA was extracted, cleaned, and concentrated as described above. The DNA concentration was determined using PicoGreen DNA quantification at the University of Minnesota Genomics Center, and 200 ng of total DNA was used for library preparation. DNA samples were barcoded with the Rapid Barcoding Kit v14 (ONT). Barcoded samples were pooled and cleaned using AMPure beads (Beckman Coulter). The pooled library was quantified with a Qubit fluorometer (Invitrogen), and the library size was determined using the Genomic DNA ScreenTape (Agilent). Rapid adaptors were added to the library immediately before loading on the MinION flow cell (ONT) and run using a GridION instrument (ONT). Portions of the prepared library were run on the MinION flow cell three times, with flow cell washing between runs, until all pores were depleted. ONT sequencing reads were collected with MinKNOW software and converted into fastq files. ONT fastq files were mapped to the *M. tuberculosis* Erdman reference genome (NC_020559.1) using Minimap2.24 in Geneious Prime software with the default settings for ONT long-read technology. The contig generated for each *eccD_5_* Tet-OFF spleen isolate was used to confirm or refute potential mutations identified by analysis of Illumina short-read sequencing data.

### Statistical analyses

Student’s unpaired *t*-test (two-tailed) was used for pairwise comparisons between strains or between conditions *in vitro* (±Atc). One-way ANOVA with Tukey’s or Dunnett’s correction applied post hoc was used for comparisons of multiple strains. Two-way ANOVA with Tukey’s correction applied post hoc was used for comparison of multiple strains and conditions. *P* values were calculated in R (CRAN r-project.org) or in GraphPad Prism (GraphPad Software, Inc). *P* values of <0.05 were considered statistically significant.

## Data Availability

All raw sequencing data from whole genome sequencing underlying the results reported are available in FASTA format at the NCBI Sequence Read Archive (BioProject no. PRJNA1348213).

## SUPPLEMENTAL MATERIAL

Supplemental Figures and Tables mbio.03500-25-s0001.pdf Figures S1-S6 and Tables S1-S3.

Table S4 mbio.03500-25-s0002.xlsx Statistical analyses of main text figures.

Table S5 mbio.03500-25-s0003.xlsx Statistical analyses of supplemental figures.

ASM does not own the copyrights to Supplemental Material that may be linked to, or accessed through, an article. The authors have granted ASM a non-exclusive, world-wide license to publish the Supplemental Material files. Please contact the corresponding author directly for reuse.

## References

[1] Bitter W, Houben ENG, Bottai D, Brodin P, Brown EJ, Cox JS, Derbyshire K, Fortune SM, Gao L-Y, Liu J, Gey van Pittius NC, Pym AS, Rubin EJ, Sherman DR, Cole ST, Brosch R. 2009. Systematic genetic nomenclature for type VII secretion systems. PLoS Pathog 5:e1000507. doi:10.1371/journal.ppat.1000507 19876390 PMC2763215

[2] Gröschel MI, Sayes F, Simeone R, Majlessi L, Brosch R. 2016. ESX secretion systems: mycobacterial evolution to counter host immunity. Nat Rev Microbiol 14:677–691. doi:10.1038/nrmicro.2016.131 27665717

[3] Pajuelo D, Tak U, Zhang L, Danilchanka O, Tischler AD, Niederweis M. 2021. Toxin secretion and trafficking by Mycobacterium tuberculosis. Nat Commun 12:6592. doi:10.1038/s41467-021-26925-1 34782620 PMC8593097

[4] Manzanillo PS, Shiloh MU, Portnoy DA, Cox JS. 2012. Mycobacterium tuberculosis activates the DNA-dependent cytosolic surveillance pathway within macrophages. Cell Host Microbe 11:469–480. doi:10.1016/j.chom.2012.03.007 22607800 PMC3662372

[5] Wong K-W, Jacobs WR Jr. 2011. Critical role for NLRP3 in necrotic death triggered by Mycobacterium tuberculosis. Cell Microbiol 13:1371–1384. doi:10.1111/j.1462-5822.2011.01625.x 21740493 PMC3257557

[6] Wassermann R, Gulen MF, Sala C, Perin SG, Lou Y, Rybniker J, Schmid-Burgk JL, Schmidt T, Hornung V, Cole ST, Ablasser A. 2015. Mycobacterium tuberculosis differentially activates cGAS- and inflammasome-dependent intracellular immune responses through ESX-1. Cell Host Microbe 17:799–810. doi:10.1016/j.chom.2015.05.003 26048138

[7] Beckwith KS, Beckwith MS, Ullmann S, Sætra RS, Kim H, Marstad A, Åsberg SE, Strand TA, Haug M, Niederweis M, Stenmark HA, Flo TH. 2020. Plasma membrane damage causes NLRP3 activation and pyroptosis during Mycobacterium tuberculosis infection. Nat Commun 11:2270. doi:10.1038/s41467-020-16143-6 32385301 PMC7210277

[8] Pajuelo D, Gonzalez-Juarbe N, Tak U, Sun J, Orihuela CJ, Niederweis M. 2018. NAD^+^ depletion triggers macrophage necroptosis, a cell death pathway exploited by Mycobacterium tuberculosis. Cell Rep 24:429–440. doi:10.1016/j.celrep.2018.06.042 29996103 PMC6136256

[9] Tufariello JM, Chapman JR, Kerantzas CA, Wong K-W, Vilchèze C, Jones CM, Cole LE, Tinaztepe E, Thompson V, Fenyö D, Niederweis M, Ueberheide B, Philips JA, Jacobs WR Jr. 2016. Separable roles for Mycobacterium tuberculosis ESX-3 effectors in iron acquisition and virulence. Proc Natl Acad Sci USA 113:E348–E357. doi:10.1073/pnas.1523321113 26729876 PMC4725510

[10] Zhang L, Hendrickson RC, Meikle V, Lefkowitz EJ, Ioerger TR, Niederweis M. 2020. Comprehensive analysis of iron utilization by Mycobacterium tuberculosis. PLoS Pathog 16:e1008337. doi:10.1371/journal.ppat.1008337 32069330 PMC7058343

[11] Portal-Celhay C, Tufariello JM, Srivastava S, Zahra A, Klevorn T, Grace PS, Mehra A, Park HS, Ernst JD, Jacobs WR Jr, Philips JA. 2016. Mycobacterium tuberculosis EsxH inhibits ESCRT-dependent CD4^+^ T-cell activation. Nat Microbiol 2:16232. doi:10.1038/nmicrobiol.2016.232 27918526 PMC5453184

[12] Mittal E, Skowyra ML, Uwase G, Tinaztepe E, Mehra A, Köster S, Hanson PI, Philips JA. 2018. Mycobacterium tuberculosis type VII secretion effectors differentially impact the ESCRT endomembrane damage response. mBio 9:e01765-18. doi:10.1128/mBio.01765-18 30482832 PMC6282207

[13] Abdallah AM, Bestebroer J, Savage NDL, de Punder K, van Zon M, Wilson L, Korbee CJ, van der Sar AM, Ottenhoff THM, van der Wel NN, Bitter W, Peters PJ. 2011. Mycobacterial secretion systems ESX-1 and ESX-5 play distinct roles in host cell death and inflammasome activation. J Immunol 187:4744–4753. doi:10.4049/jimmunol.1101457 21957139

[14] Beckham KSH, Ritter C, Chojnowski G, Ziemianowicz DS, Mullapudi E, Rettel M, Savitski MM, Mortensen SA, Kosinski J, Wilmanns M. 2021. Structure of the mycobacterial ESX-5 type VII secretion system pore complex. Sci Adv 7:eabg9923. doi:10.1126/sciadv.abg9923 34172453 PMC8232910

[15] Bunduc CM, Fahrenkamp D, Wald J, Ummels R, Bitter W, Houben ENG, Marlovits TC. 2021. Structure and dynamics of the ESX-5 type VII secretion system of Mycobacterium tuberculosis. Nature 593:445–448. doi:10.1038/s41586-021-03517-z 33981042 PMC8131196

[16] DeJesus MA, Gerrick ER, Xu W, Park SW, Long JE, Boutte CC, Rubin EJ, Schnappinger D, Ehrt S, Fortune SM, Sassetti CM, Ioerger TR. 2017. Comprehensive essentiality analysis of the Mycobacterium tuberculosis genome via saturating transposon mutagenesis. mBio 8:e02133-16. doi:10.1128/mBio.02133-16 28096490 PMC5241402

[17] Carey AF, Rock JM, Krieger IV, Chase MR, Fernandez-Suarez M, Gagneux S, Sacchettini JC, Ioerger TR, Fortune SM. 2018. TnSeq of Mycobacterium tuberculosis clinical isolates reveals strain-specific antibiotic liabilities. PLoS Pathog 14:e1006939. doi:10.1371/journal.ppat.1006939 29505613 PMC5854444

[18] Di Luca M, Bottai D, Batoni G, Orgeur M, Aulicino A, Counoupas C, Campa M, Brosch R, Esin S. 2012. The ESX-5 associated eccB5-eccC5 locus is essential for Mycobacterium tuberculosis viability. PLoS One 7:e52059. doi:10.1371/journal.pone.0052059 23284869 PMC3524121

[19] White DW, Elliott SR, Odean E, Bemis LT, Tischler AD. 2018. Mycobacterium tuberculosis Pst/SenX3-RegX3 regulates membrane vesicle production independently of ESX-5 activity. mBio 9:e00778-18. doi:10.1128/mBio.00778-18 29895636 PMC6016242

[20] Ates LS, Ummels R, Commandeur S, van de Weerd R, Sparrius M, Weerdenburg E, Alber M, Kalscheuer R, Piersma SR, Abdallah AM, Abd El Ghany M, Abdel-Haleem AM, Pain A, Jiménez CR, Bitter W, Houben ENG. 2015. Essential role of the ESX-5 secretion system in outer membrane permeability of pathogenic mycobacteria. PLoS Genet 11:e1005190. doi:10.1371/journal.pgen.1005190 25938982 PMC4418733

[21] Bottai D, Di Luca M, Majlessi L, Frigui W, Simeone R, Sayes F, Bitter W, Brennan MJ, Leclerc C, Batoni G, Campa M, Brosch R, Esin S. 2012. Disruption of the ESX-5 system of Mycobacterium tuberculosis causes loss of PPE protein secretion, reduction of cell wall integrity and strong attenuation. Mol Microbiol 83:1195–1209. doi:10.1111/j.1365-2958.2012.08001.x 22340629

[22] Tiwari S, Dutt TS, Chen B, Chen M, Kim J, Dai AZ, Lukose R, Shanley C, Fox A, Karger BR, Porcelli SA, Chan J, Podell BK, Obregon-Henao A, Orme IM, Jacobs WR Jr, Henao-Tamayo M. 2020. BCG-prime and boost with ESX-5 secretion system deletion mutant leads to better protection against clinical strains of Mycobacterium tuberculosis. Vaccine (Auckl) 38:7156–7165. doi:10.1016/j.vaccine.2020.08.004 PMC7755135 32978002

[23] Koleske B, Rajagopalan S, Schill C, Lun S, Vilchèze C, Das L, Gupta M, Martinez-Martinez YB, Bishai WR, Jacobs WR Jr. 2025. Loss of the ESX-5 secretion locus in Mycobacterium tuberculosis reshapes the mycomembrane and enhances ESX-1 substrate secretion. Proc Natl Acad Sci USA 122:e2509997122. doi:10.1073/pnas.2509997122 40901885 PMC12435201

[24] Cox JS, Chen B, McNeil M, Jacobs WR Jr. 1999. Complex lipid determines tissue-specific replication of Mycobacterium tuberculosis in mice. Nature 402:79–83. doi:10.1038/47042 10573420

[25] Kirksey MA, Tischler AD, Siméone R, Hisert KB, Uplekar S, Guilhot C, McKinney JD. 2011. Spontaneous phthiocerol dimycocerosate-deficient variants of Mycobacterium tuberculosis are susceptible to gamma interferon-mediated immunity. Infect Immun 79:2829–2838. doi:10.1128/IAI.00097-11 21576344 PMC3191967

[26] Stahl C, Kubetzko S, Kaps I, Seeber S, Engelhardt H, Niederweis M. 2001. MspA provides the main hydrophilic pathway through the cell wall of Mycobacterium smegmatis. Mol Microbiol 40:451–464. doi:10.1046/j.1365-2958.2001.02394.x 11309127

[27] Stephan J, Bender J, Wolschendorf F, Hoffmann C, Roth E, Mailänder C, Engelhardt H, Niederweis M. 2005. The growth rate of Mycobacterium smegmatis depends on sufficient porin-mediated influx of nutrients. Mol Microbiol 58:714–730. doi:10.1111/j.1365-2958.2005.04878.x 16238622

[28] Wang Q, Boshoff HIM, Harrison JR, Ray PC, Green SR, Wyatt PG, Barry CE 3rd. 2020. PE/PPE proteins mediate nutrient transport across the outer membrane of Mycobacterium tuberculosis. Science 367:1147–1151. doi:10.1126/science.aav5912 32139546 PMC11036889

[29] Camacho LR, Constant P, Raynaud C, Laneelle MA, Triccas JA, Gicquel B, Daffe M, Guilhot C. 2001. Analysis of the phthiocerol dimycocerosate locus of Mycobacterium tuberculosis. Evidence that this lipid is involved in the cell wall permeability barrier. J Biol Chem 276:19845–19854. doi:10.1074/jbc.M100662200 11279114

[30] Soetaert K, Rens C, Wang X-M, De Bruyn J, Lanéelle M-A, Laval F, Lemassu A, Daffé M, Bifani P, Fontaine V, Lefèvre P. 2015. Increased vancomycin susceptibility in mycobacteria: a new approach to identify synergistic activity against multidrug-resistant mycobacteria. Antimicrob Agents Chemother 59:5057–5060. doi:10.1128/AAC.04856-14 26033733 PMC4505240

[31] Mulholland CV, Wiggins TJ, Cui J, Vilchèze C, Rajagopalan S, Shultis MW, Reyes-Fernández EZ, Jacobs WR Jr, Berney M. 2024. Propionate prevents loss of the PDIM virulence lipid in Mycobacterium tuberculosis. Nat Microbiol 9:1607–1618. doi:10.1038/s41564-024-01697-8 38740932 PMC11253637

[32] Abdallah AM, Verboom T, Weerdenburg EM, Gey van Pittius NC, Mahasha PW, Jiménez C, Parra M, Cadieux N, Brennan MJ, Appelmelk BJ, Bitter W. 2009. PPE and PE_PGRS proteins of Mycobacterium marinum are transported via the type VII secretion system ESX-5. Mol Microbiol 73:329–340. doi:10.1111/j.1365-2958.2009.06783.x 19602152

[33] Gey van Pittius NC, Sampson SL, Lee H, Kim Y, van Helden PD, Warren RM. 2006. Evolution and expansion of the Mycobacterium tuberculosis PE and PPE multigene families and their association with the duplication of the ESAT-6 (esx) gene cluster regions. BMC Evol Biol 6:95. doi:10.1186/1471-2148-6-95 17105670 PMC1660551

[34] Fishbein S, van Wyk N, Warren RM, Sampson SL. 2015. Phylogeny to function: PE/PPE protein evolution and impact on Mycobacterium tuberculosis pathogenicity. Mol Microbiol 96:901–916. doi:10.1111/mmi.12981 25727695

[35] Cole ST, Brosch R, Parkhill J, Garnier T, Churcher C, Harris D, Gordon SV, Eiglmeier K, Gas S, Barry CE III, et al.. 1998. Deciphering the biology of Mycobacterium tuberculosis from the complete genome sequence. Nature 393:537–544. doi:10.1038/31159 9634230

[36] Sayes F, Sun L, Di Luca M, Simeone R, Degaiffier N, Fiette L, Esin S, Brosch R, Bottai D, Leclerc C, Majlessi L. 2012. Strong immunogenicity and cross-reactivity of Mycobacterium tuberculosis ESX-5 type VII secretion: encoded PE-PPE proteins predicts vaccine potential. Cell Host Microbe 11:352–363. doi:10.1016/j.chom.2012.03.003 22520463

[37] Mitra A, Speer A, Lin K, Ehrt S, Niederweis M. 2017. PPE surface proteins are required for heme utilization by Mycobacterium tuberculosis. mBio 8:e01720-16. doi:10.1128/mBio.01720-16 28119467 PMC5263243

[38] Tullius MV, Nava S, Horwitz MA. 2019. PPE37 is essential for Mycobacterium tuberculosis heme-iron acquisition (HIA), and a defective PPE37 in Mycobacterium bovis BCG prevents HIA. Infect Immun 87:e00540-18. doi:10.1128/IAI.00540-18 PMC6346139 30455201

[39] Boradia V, Frando A, Grundner C. 2022. The Mycobacterium tuberculosis PE15/PPE20 complex transports calcium across the outer membrane. PLoS Biol 20:e3001906. doi:10.1371/journal.pbio.3001906 36441815 PMC9731449

[40] Korycka-Machała M, Pawełczyk J, Borówka P, Dziadek B, Brzostek A, Kawka M, Bekier A, Rykowski S, Olejniczak AB, Strapagiel D, Witczak Z, Dziadek J. 2020. PPE51 is involved in the uptake of disaccharides by Mycobacterium tuberculosis. Cells 9:603. doi:10.3390/cells9030603 32138343 PMC7140425

[41] Sankey N, Merrick H, Singh P, Rogers J, Reddi A, Hartson SD, Mitra A. 2023. Role of the Mycobacterium tuberculosis ESX-4 secretion system in heme iron utilization and pore formation by PPE proteins. mSphere 8:e0057322. doi:10.1128/msphere.00573-22 36749044 PMC10117145

[42] Babu Sait MR, Koliwer-Brandl H, Stewart JA, Swarts BM, Jacobsen M, Ioerger TR, Kalscheuer R. 2022. PPE51 mediates uptake of trehalose across the mycomembrane of Mycobacterium tuberculosis. Sci Rep 12:2097. doi:10.1038/s41598-022-06109-7 35136132 PMC8826857

[43] Dechow SJ, Baker JJ, Murto M, Abramovitch RB. 2022. ppe51 variants enable growth of Mycobacterium tuberculosis at acidic pH by selectively promoting glycerol uptake. J Bacteriol 204:e0021222. doi:10.1128/jb.00212-22 36226966 PMC9664963

[44] Jones CM, Niederweis M. 2010. Role of porins in iron uptake by Mycobacterium smegmatis. J Bacteriol 192:6411–6417. doi:10.1128/JB.00986-10 20952578 PMC3008526

[45] Speer A, Rowland JL, Haeili M, Niederweis M, Wolschendorf F. 2013. Porins increase copper susceptibility of Mycobacterium tuberculosis. J Bacteriol 195:5133–5140. doi:10.1128/JB.00763-13 24013632 PMC3811576

[46] Botella H, Peyron P, Levillain F, Poincloux R, Poquet Y, Brandli I, Wang C, Tailleux L, Tilleul S, Charrière GM, Waddell SJ, Foti M, Lugo-Villarino G, Gao Q, Maridonneau-Parini I, Butcher PD, Castagnoli PR, Gicquel B, de Chastellier C, Neyrolles O. 2011. Mycobacterial p(1)-type ATPases mediate resistance to zinc poisoning in human macrophages. Cell Host Microbe 10:248–259. doi:10.1016/j.chom.2011.08.006 21925112 PMC3221041

[47] Wolschendorf F, Ackart D, Shrestha TB, Hascall-Dove L, Nolan S, Lamichhane G, Wang Y, Bossmann SH, Basaraba RJ, Niederweis M. 2011. Copper resistance is essential for virulence of Mycobacterium tuberculosis. Proc Natl Acad Sci USA 108:1621–1626. doi:10.1073/pnas.1009261108 21205886 PMC3029754

[48] Dow A, Sule P, O’Donnell TJ, Burger A, Mattila JT, Antonio B, Vergara K, Marcantonio E, Adams LG, James N, Williams PG, Cirillo JD, Prisic S. 2021. Zinc limitation triggers anticipatory adaptations in Mycobacterium tuberculosis. PLoS Pathog 17:e1009570. doi:10.1371/journal.ppat.1009570 33989345 PMC8121289

[49] Marrero J, Rhee KY, Schnappinger D, Pethe K, Ehrt S. 2010. Gluconeogenic carbon flow of tricarboxylic acid cycle intermediates is critical for Mycobacterium tuberculosis to establish and maintain infection. Proc Natl Acad Sci USA 107:9819–9824. doi:10.1073/pnas.1000715107 20439709 PMC2906907

[50] Gengenbacher M, Zimmerman MD, Sarathy JP, Kaya F, Wang H, Mina M, Carter C, Hossen MA, Su H, Trujillo C, Ehrt S, Schnappinger D, Dartois V. 2020. Tissue distribution of doxycycline in animal models of tuberculosis. Antimicrob Agents Chemother 64:e02479-19. doi:10.1128/AAC.02479-19 32041718 PMC7179585

[51] Gill WP, Harik NS, Whiddon MR, Liao RP, Mittler JE, Sherman DR. 2009. A replication clock for Mycobacterium tuberculosis. Nat Med 15:211–214. doi:10.1038/nm.1915 19182798 PMC2779834

[52] Charitou V, Izquierdo Lafuente B, Habjan E, Kuijl C, Willemse JJ, Bitter W, Speer A. 2025. PPE51 modulates membrane integrity in Mycobacterium marinum. mBio 16:e0104425. doi:10.1128/mbio.01044-25 40980894 PMC12607722

[53] Weerdenburg EM, Abdallah AM, Mitra S, de Punder K, van der Wel NN, Bird S, Appelmelk BJ, Bitter W, van der Sar AM. 2012. ESX-5-deficient Mycobacterium marinum is hypervirulent in adult zebrafish. Cell Microbiol 14:728–739. doi:10.1111/j.1462-5822.2012.01755.x 22256857

[54] Wagner D, Maser J, Lai B, Cai Z, Barry CE 3rd, Höner Zu Bentrup K, Russell DG, Bermudez LE. 2005. Elemental analysis of Mycobacterium avium-, Mycobacterium tuberculosis-, and Mycobacterium smegmatis-containing phagosomes indicates pathogen-induced microenvironments within the host cell’s endosomal system. J Immunol 174:1491–1500. doi:10.4049/jimmunol.174.3.1491 15661908

[55] Ward SK, Abomoelak B, Hoye EA, Steinberg H, Talaat AM. 2010. CtpV: a putative copper exporter required for full virulence of Mycobacterium tuberculosis. Mol Microbiol 77:1096–1110. doi:10.1111/j.1365-2958.2010.07273.x 20624225 PMC2965804

[56] León-Torres A, Novoa-Aponte L, Soto CY. 2015. CtpA, a putative Mycobacterium tuberculosis P-type ATPase, is stimulated by copper (I) in the mycobacterial plasma membrane. Biometals 28:713–724. doi:10.1007/s10534-015-9860-x 25967101

[57] Szczepanik W, Czarny A, Zaczyńska E, Jeżowska-Bojczuk M. 2004. Preferences of kanamycin A towards copper(II). Effect of the resulting complexes on immunological mediators production by human leukocytes. J Inorg Biochem 98:245–253. doi:10.1016/j.jinorgbio.2003.10.013 14729305

[58] Strong EJ, Wang J, Ng TW, Porcelli SA, Lee S. 2022. Mycobacterium tuberculosis PPE51 inhibits autophagy by suppressing Toll-like receptor 2-dependent signaling. mBio 13:e0297421. doi:10.1128/mbio.02974-21 35467412 PMC9239179

[59] Pethe K, Sequeira PC, Agarwalla S, Rhee K, Kuhen K, Phong WY, Patel V, Beer D, Walker JR, Duraiswamy J, et al.. 2010. A chemical genetic screen in Mycobacterium tuberculosis identifies carbon-source-dependent growth inhibitors devoid of in vivo efficacy. Nat Commun 1:57. doi:10.1038/ncomms1060 20975714 PMC3220188

[60] Marrero J, Trujillo C, Rhee KY, Ehrt S. 2013. Glucose phosphorylation is required for Mycobacterium tuberculosis persistence in mice. PLoS Pathog 9:e1003116. doi:10.1371/journal.ppat.1003116 23326232 PMC3542180

[61] Wilburn KM, Fieweger RA, VanderVen BC. 2018. Cholesterol and fatty acids grease the wheels of Mycobacterium tuberculosis pathogenesis. Pathog Dis 76:fty021. doi:10.1093/femspd/fty021 29718271 PMC6251666

[62] Nazarova EV, Montague CR, La T, Wilburn KM, Sukumar N, Lee W, Caldwell S, Russell DG, VanderVen BC. 2017. Rv3723/LucA coordinates fatty acid and cholesterol uptake in Mycobacterium tuberculosis. eLife 6:e26969. doi:10.7554/eLife.26969 28708968 PMC5487216

[63] Deb C, Daniel J, Sirakova TD, Abomoelak B, Dubey VS, Kolattukudy PE. 2006. A novel lipase belonging to the hormone-sensitive lipase family induced under starvation to utilize stored triacylglycerol in Mycobacterium tuberculosis. J Biol Chem 281:3866–3875. doi:10.1074/jbc.M505556200 16354661 PMC1523426

[64] Sultana R, Vemula MH, Banerjee S, Guruprasad L. 2013. The PE16 (Rv1430) of Mycobacterium tuberculosis is an esterase belonging to serine hydrolase superfamily of proteins. PLoS One 8:e55320. doi:10.1371/journal.pone.0055320 23383323 PMC3562317

[65] Sultana R, Tanneeru K, Guruprasad L. 2011. The PE-PPE domain in mycobacterium reveals a serine α/β hydrolase fold and function: an in-silico analysis. PLoS One 6:e16745. doi:10.1371/journal.pone.0016745 21347309 PMC3037379

[66] Stanley SA, Johndrow JE, Manzanillo P, Cox JS. 2007. The type I IFN response to infection with Mycobacterium tuberculosis requires ESX-1-mediated secretion and contributes to pathogenesis. J Immunol 178:3143–3152. doi:10.4049/jimmunol.178.5.3143 17312162

[67] Fortune SM, Jaeger A, Sarracino DA, Chase MR, Sassetti CM, Sherman DR, Bloom BR, Rubin EJ. 2005. Mutually dependent secretion of proteins required for mycobacterial virulence. Proc Natl Acad Sci USA 102:10676–10681. doi:10.1073/pnas.0504922102 16030141 PMC1176248

[68] Lewis KN, Liao R, Guinn KM, Hickey MJ, Smith S, Behr MA, Sherman DR. 2003. Deletion of RD1 from Mycobacterium tuberculosis mimics bacille Calmette-Guérin attenuation. J Infect Dis 187:117–123. doi:10.1086/345862 12508154 PMC1458498

[69] Cohen SB, Gern BH, Delahaye JL, Adams KN, Plumlee CR, Winkler JK, Sherman DR, Gerner MY, Urdahl KB. 2018. Alveolar macrophages provide an early Mycobacterium tuberculosis niche and initiate dissemination. Cell Host Microbe 24:439–446. doi:10.1016/j.chom.2018.08.001 30146391 PMC6152889

[70] Murphy J, Summer R, Wilson AA, Kotton DN, Fine A. 2008. The prolonged life-span of alveolar macrophages. Am J Respir Cell Mol Biol 38:380–385. doi:10.1165/rcmb.2007-0224RC 18192503 PMC2274942

[71] Reiley WW, Calayag MD, Wittmer ST, Huntington JL, Pearl JE, Fountain JJ, Martino CA, Roberts AD, Cooper AM, Winslow GM, Woodland DL. 2008. ESAT-6-specific CD4 T cell responses to aerosol Mycobacterium tuberculosis infection are initiated in the mediastinal lymph nodes. Proc Natl Acad Sci USA 105:10961–10966. doi:10.1073/pnas.0801496105 18667699 PMC2504808

[72] Rybniker J, Chen JM, Sala C, Hartkoorn RC, Vocat A, Benjak A, Boy-Röttger S, Zhang M, Székely R, Greff Z, Orfi L, Szabadkai I, Pató J, Kéri G, Cole ST. 2014. Anticytolytic screen identifies inhibitors of mycobacterial virulence protein secretion. Cell Host Microbe 16:538–548. doi:10.1016/j.chom.2014.09.008 25299337

[73] Jia P, Zhang Y, Xu J, Zhu M, Peng S, Zhang Y, Zhao J, Li X, Mi K, Yan D, Wang Y, Yu L, Lu Y, Shi H, Cen S. 2022. IMB-BZ as an inhibitor targeting ESX-1 secretion system to control mycobacterial infection. J Infect Dis 225:608–616. doi:10.1093/infdis/jiab486 34558604

[74] Gries R, Chhen J, van Gumpel E, Theobald SJ, Sonnenkalb L, Utpatel C, Metzen F, Koch M, Dallenga T, Djaout K, Baulard A, Dal Molin M, Rybniker J. 2024. Discovery of dual-active ethionamide boosters inhibiting the Mycobacterium tuberculosis ESX-1 secretion system. Cell Chem Biol 31:699–711. doi:10.1016/j.chembiol.2023.12.007 38181799

[75] Ho VQT, Rong MK, Habjan E, Bommer SD, Pham TV, Piersma SR, Bitter W, Ruijter E, Speer A. 2023. Dysregulation of Mycobacterium marinum ESX-5 secretion by novel 1,2,4-oxadiazoles. Biomolecules 13:211. doi:10.3390/biom13020211 36830581 PMC9953084

[76] Beckham KSH, Ciccarelli L, Bunduc CM, Mertens HDT, Ummels R, Lugmayr W, Mayr J, Rettel M, Savitski MM, Svergun DI, Bitter W, Wilmanns M, Marlovits TC, Parret AHA, Houben ENG. 2017. Structure of the mycobacterial ESX-5 type VII secretion system membrane complex by single-particle analysis. Nat Microbiol 2:17047. doi:10.1038/nmicrobiol.2017.47 28394313

[77] Pandey AK, Sassetti CM. 2008. Mycobacterial persistence requires the utilization of host cholesterol. Proc Natl Acad Sci USA 105:4376–4380. doi:10.1073/pnas.0711159105 18334639 PMC2393810

[78] Tischler AD, Leistikow RL, Kirksey MA, Voskuil MI, McKinney JD. 2013. Mycobacterium tuberculosis requires phosphate-responsive gene regulation to resist host immunity. Infect Immun 81:317–328. doi:10.1128/IAI.01136-12 23132496 PMC3536151

[79] Elliott SR, Tischler AD. 2016. Phosphate starvation: a novel signal that triggers ESX-5 secretion in Mycobacterium tuberculosis. Mol Microbiol 100:510–526. doi:10.1111/mmi.13332 26800324 PMC4863468

[80] Kamariza M, Shieh P, Bertozzi CR. 2018. Imaging mycobacterial trehalose glycolipids. Methods Enzymol 598:355–369. doi:10.1016/bs.mie.2017.09.002 29306442 PMC6038140

[81] Block AM, Namugenyi SB, Palani NP, Brokaw AM, Zhang L, Beckman KB, Tischler AD. 2023. Mycobacterium tuberculosis requires the outer membrane lipid phthiocerol dimycocerosate for starvation-induced antibiotic tolerance. mSystems 8:e0069922. doi:10.1128/msystems.00699-22 36598240 PMC9948706

[82] Ramakrishnan P, Aagesen AM, McKinney JD, Tischler AD. 2016. Mycobacterium tuberculosis resists stress by regulating PE19 expression. Infect Immun 84:735–746. doi:10.1128/IAI.00942-15 PMC4771341 26712204

[83] Larsen MH, Biermann K, Tandberg S, Hsu T, Jacobs, WR Jr. 2007. Genetic manipulation of Mycobacterium tuberculosis. CP Microbiology 6. doi:10.1002/9780471729259.mc10a02s6 18770603

[84] National Research Council. 2011. Guide for the care and use of laboratory animals. 8th ed. National Academies Press, Washington, DC.

