# Supplementary material for: The Mycobacterium tuberculosis ESX-5 secretion system enables carbon source utilization and growth in mice
Source: mBio. 2026 Jan 30;17(3):e03500-25. doi: 10.1128/mbio.03500-25 (PMC12977502; doi:10.1128/mbio.03500-25)
Supplement: Supplemental Figures and Tables — Figures S1-S6 and Tables S1-S3. [file mbio.03500-25-s0001.pdf]

Supplementary Figures S1-S6 and Tables S1-S3

The *Mycobacterium tuberculosis* ESX-5 secretion system enables carbon source utilization and growth in mice

Alisha M. Block[1], Rashmi Ravindran Nair[2], Virginia Meikle[2], Parker C. Wiegert[1], Dylan W. White[1], Leanne Zhang[1], Michael Niederweis[2] and Anna D. Tischler[1#]

[1]Department of Microbiology and Immunology, University of Minnesota, Minneapolis, MN 55455

[2]Department of Microbiology, University of Alabama at Birmingham, Birmingham, AL 35294

# Corresponding author: Anna D. Tischler, tischler@umn.edu

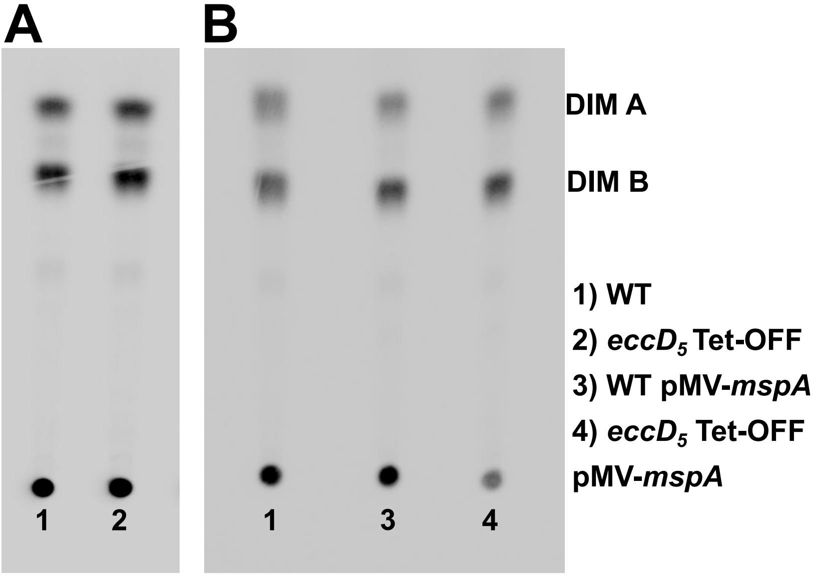

Fig S1. ***M. tuberculosis eccD$_5$*** **Tet-OFF strains produce phthiocerol dimycocerosate (PDIM).** The indicated strains were grown in complete 7H9 medium and labeled for 48 hr with 10 μCi ^{14}C propionate prior to extraction of lipids and analysis of PDIM production by thin-layer chromatography. The DIM A and DIM B forms of PDIM are indicated. (**A**) WT Erdman and *eccD$_5$* Tet-OFF. (**B**) WT Erdman, *eccD$_5$* Tet-OFF pMV261 and *eccD$_5$* Tet-OFF pMV-*mspA*.

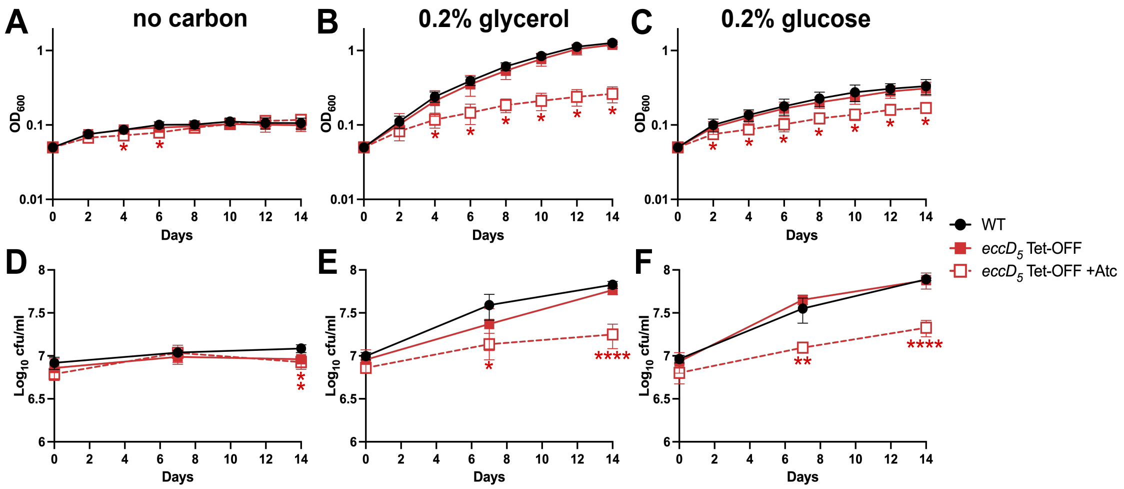

Fig S2. *M. tuberculosis* requires ESX-5 for *in vitro* growth in minimal medium with glycerol or glucose. WT Erdman and *eccD*$_5$ Tet-OFF were grown in complete Middlebrook 7H9 ± 100 ng/ml Atc to mid-exponential phase, then washed and diluted to OD_{600} = 0.05 in minimal salts medium with 0.05% tyloxapol and no added carbon (**A, D**), 0.2% glycerol (**B, E**), or 0.2% glucose (**C, F**). Fresh Atc (100 ng/ml) was added to +Atc cultures every 7 days. Growth was monitored by OD_{600} measurements every 2 days (**A-C**) or by plating serially diluted cultures on Middlebrook 7H10 agar every 7 days (**D-F**). Data are means ± standard deviations of 4 biological replicates from 2 independent experiments. Statistical analyses were performed to compare *eccD*$_5$ Tet-OFF ± Atc to WT by one-way ANOVA with Dunnett's correction (**p*<0.05, ***p*<0.01, *****p*<0.0001). Complete results of statistical analyses are in **Table S5**.

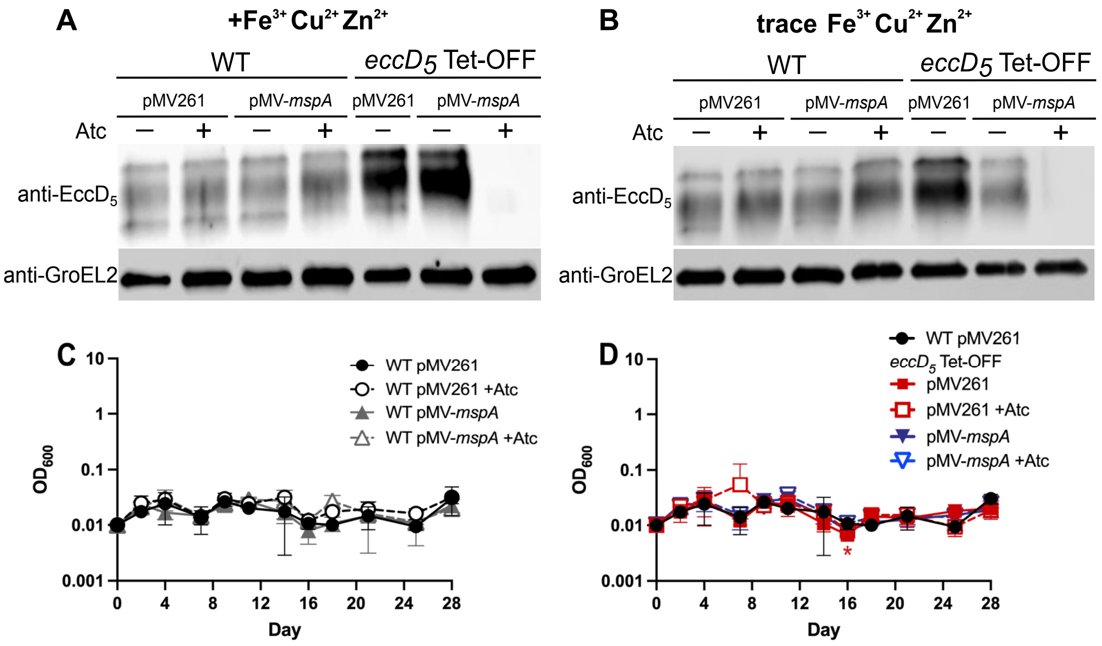

Fig S3. Western blotting and 7H9 with no added carbon growth curve controls for *M. tuberculosis* strains expressing MspA. (A, B) Western blots to confirm $EccD_5$ depletion. Whole cell lysates were prepared from cultures grown in 7H9 + 0.5% glycerol (**A**) or in 7H9 with trace Fe^{3+}, Cu^{2+} and Zn^{2+} + 0.5% glycerol (**B**) that are shown in Fig 2. $EccD_5$ and GroEL2 were detected in 5.8 μg total protein (**A**) or 10 μg total protein (**B**) by Western blotting. (**C,D**) The indicated strains were grown in complete Middlebrook 7H9 ± 100 ng/ml Atc to mid-exponential phase, then washed and diluted in triplicate to OD_{600} = 0.01 in home-made Middlebrook 7H9 with trace Fe^{3+} and Cu^{2+} with 0.01% tyloxapol and no added carbon ± 100 ng/ml anhydrotetracycline (Atc). Growth was monitored by OD_{600} measurements every 2-3 days. Fresh Atc (100 ng/ml) was added to +Atc cultures every 7 days. Data are means ± standard deviations. Statistical analyses were done to compare each strain and condition to the WT pMV261 untreated control (**C**) or to the *$eccD_5$* Tet-OFF pMV261 untreated control (**D**) (*p<0.05; two-way ANOVA with a simple effects model and Dunnett's correction). Complete results of statistical analyses are in **Table S5**.

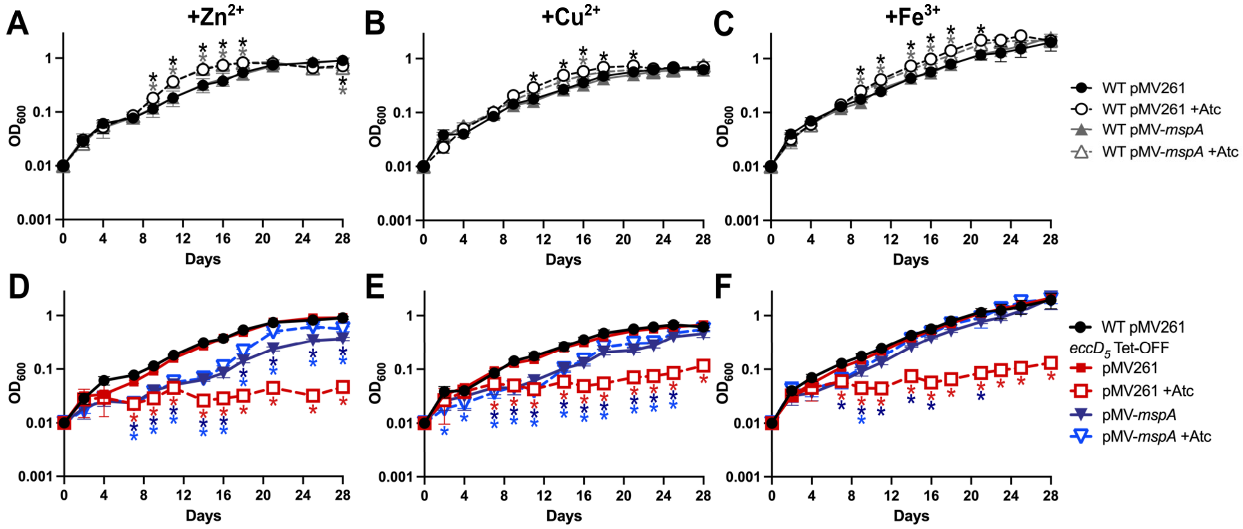

Fig S4. *M. tuberculosis eccD*$_5$ Tet-OFF expressing MspA experiences Zn^{2+} and Cu^{2+} but not Fe^{3+} toxicity. The indicated strains were grown in complete Middlebrook 7H9 ± 100 ng/ml Atc to mid-exponential phase, then washed and diluted in triplicate to OD_{600} = 0.01 in self-made trace heavy metals Middlebrook 7H9 with 0.01% tyloxapol and 0.5% glycerol ± 100 ng/ml anhydrotetracycline (Atc). Zn^{2+} (**A,D**) Cu^{2+} (**B,E**), or Fe^{3+} (**C,F**) was added to cultures at the concentration found in standard Middlebrook 7H9 medium. Growth was monitored by OD_{600} measurements every 2-3 days. Fresh Atc (100 ng/ml) was added to +Atc cultures every 7 days. Data are means ± standard deviations. Statistical analyses were done to compare each strain and condition to the WT pMV261 untreated control (**A-C**) or to compare each strain and condition to the *eccD*$_5$ Tet-OFF pMV261 untreated control (**D-F**) (**p*<0.05; two-way ANOVA with a simple effects model and Dunnett's correction). Complete results of statistical analyses are in **Table S5**.

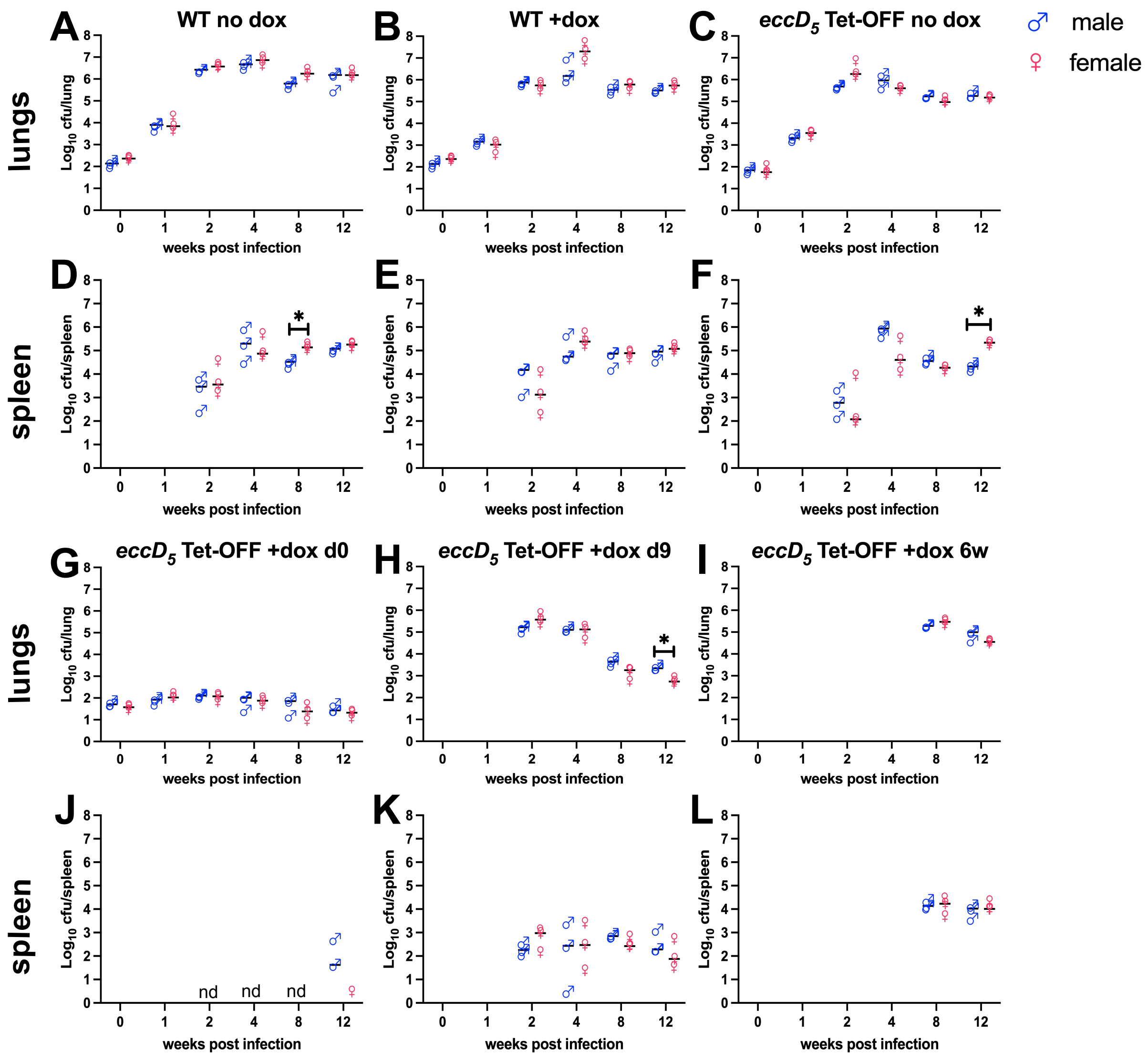

Fig S5. ***M. tuberculosis*** **lung and spleen burdens from individual mice, separated by sex.** Data from C57BL/6 mice that were infected by aerosol with WT Erdman (**A, B, D, E**) or *eccD*$_5$ Tet-OFF (**C, F, G-L**) and treated with doxycycline (2,000 ppm in chow) starting at the indicated time points, from Fig 5. *M. tuberculosis* CFU in lung (**A-C, G-I**) and spleen (**D-F, J-L**) tissues were determined by plating and data from male and female mice (n=3 each) are shown separately. Lines indicate the mean. In **J**, n.d. indicates none detected in either male or female mice (detection limit = 3 CFU). Asterisks indicate statistically significant differences between male and female mice in each experimental group (*p<0.05; unpaired t-test). Complete results of statistical analyses are in **Table S5**.

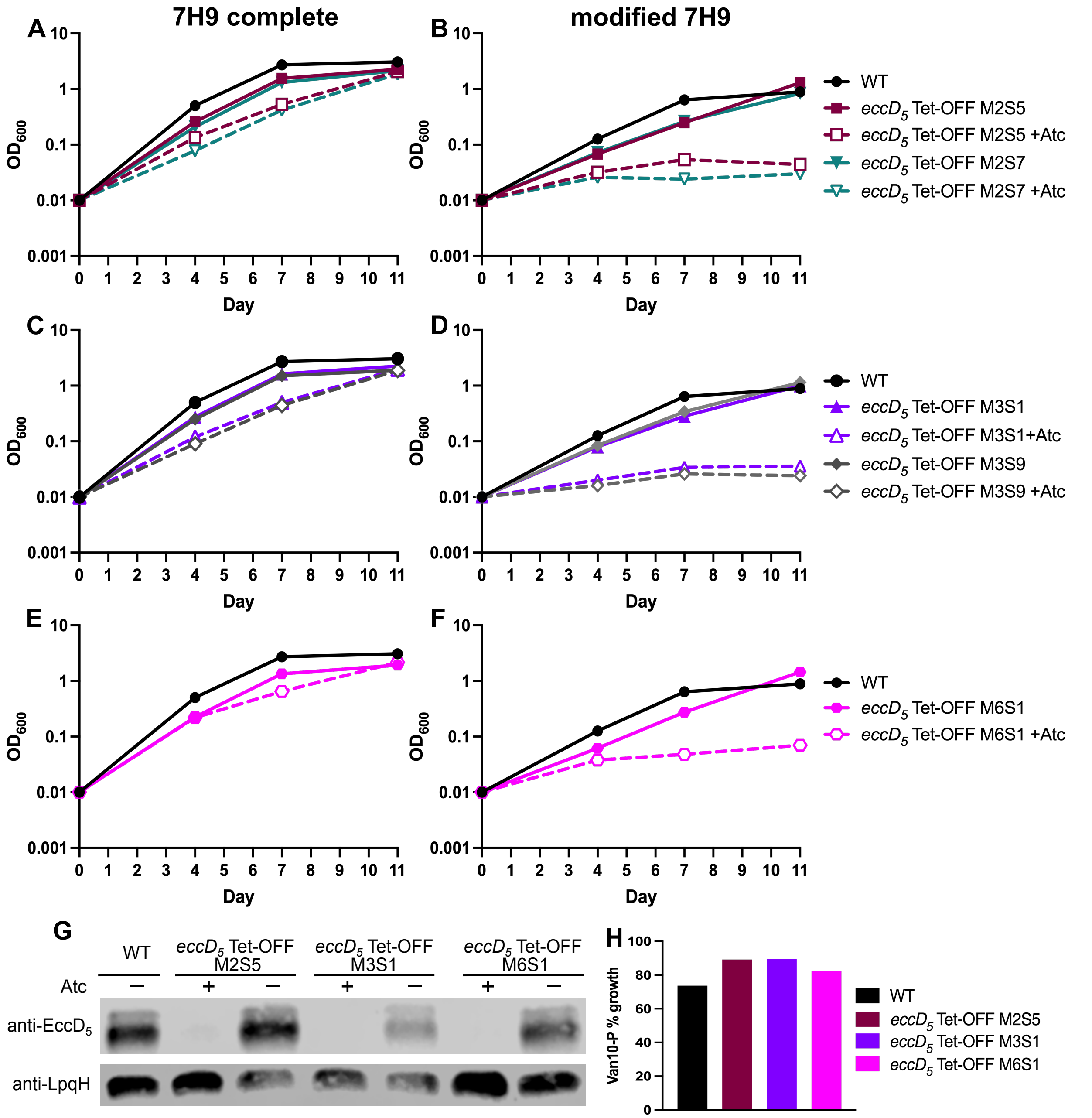

Fig S6. *M. tuberculosis eccD₅* Tet-OFF isolates recovered from spleens of dox-treated mice at 12 weeks post-infection retain Atc-repressible $EccD_5$ expression. **(A-F)** *M. tuberculosis eccD₅* Tet-OFF mouse spleen isolates or WT control were grown in complete Middlebrook 7H9 ± 100 ng/ml Atc to mid-exponential phase, then washed and diluted to OD_{600} = 0.01 in complete 7H9 (**A, C, E**), or modified 7H9 with 0.01% tyloxapol +0.5% glycerol +0.2% glucose (**B, D, F**) ± 100 ng/ml anhydrotetracycline (Atc). Growth was monitored by OD_{600} measurements every 3-4 days. Fresh Atc (100 ng/ml) was added to +Atc cultures at day 7. (**G**) *M. tuberculosis eccD₅* Tet-OFF mouse spleen isolates and the WT control were grown in complete 7H9 ± 100 ng/ml Atc and whole cell lysates were prepared. $EccD_5$ and LpqH were detected in 11.9 μg of total protein by Western blotting. (**H**) Vancomycin susceptibility in the presence of propionate quantified by Van10-P assay. Van10-P % growth is [(OD_{600} 10 μg Van)/(OD_{600} 0 μg Van)] x100.

Table S1. ***Mycobacterium tuberculosis*** **strains used in this study.**

Strain name	Genotype	Source
WT *M. tuberculosis* Erdman	wild type	Lab strain
eccD$_5$ Tet-OFF	Δ*eccD*$_5$ pGMCH-*eccD*$_5$ Tet-OFF, Hyg^R	(1)
WT pMV261	pMV261, Kan^R	This study
WT pMV-*mspA*	pMV261-*mspA*, Kan^R	This study
eccD$_5$ Tet-OFF pMV261	Δ*eccD*$_5$ pGMCH-*eccD*$_5$ Tet-OFF pMV261, Hyg^R Kan^R	This study
eccD$_5$ Tet-OFF pMV-*mspA*	Δ*eccD*$_5$ pGMCH-*eccD*$_5$ Tet-OFF pMV-*mspA*, Hyg^R Kan^R	This study
WT pMV-*ppe51-His6*	pMV-*ppe51-His6*, Kan^R	This study
eccD$_5$ Tet-OFF pMV-*ppe51-His6*	Δ*eccD*$_5$ pGMCH-*eccD*$_5$ Tet-OFF pMV-*ppe51-His6*, Hyg^R Kan^R	This study

Table S2. Plasmids used in this study.

Plasmid name	Description	Source
pMV261	episomal plasmid with constitutive P_{hsp60} promoter, Kan^R	(2)
	episomal plasmid with *M. smegmatis mspA* under the P_{smyc} optimal mycobacterial promoter	(3)
pMV-*mspA*	episomal plasmid with *M. smegmatis mspA* under the P_{smyc} optimal mycobacterial promoter, Kan^R	This study
pMV306-HNK-*ppe51-His6*	L5 *attB* integrating plasmid with *ppe51-His6* under the constitutive P_{hsp60} promoter, Kan^R	(4)
pMV-*ppe51-His6*	episomal plasmid with *ppe51-His6* under the constitutive P_{hsp60} promoter, Kan^R	This study

Table S3. Oligonucleotide primers used in this study.

Primer name	Sequence 5'-3'	Use
mspA_F	gagcacaggcacctctcac	Check for pMV-*mspA* in *M. tuberculosis*
mspA_R	gacgtcgaccgagaacgttg	Check for pMV-*mspA* in *M. tuberculosis*
pMV361_seqF	cagcgaggacaacttgagcc	Check for pMV-*ppe51-His6* in *M. tuberculosis*
ppe51R	ctcgcgagcaccgtgttg	Check for pMV-*ppe51-His6* in *M. tuberculosis*

References

1. White DW, Elliott SR, Odean E, Bemis LT, Tischler AD. 2018. *Mycobacterium tuberculosis* Pst/SenX3-RegX3 regulates membrane vesicle production independently of ESX-5 activity. mBio 9:e00778-18.
2. Stover CK, de la Cruz VF, Fuerst TR, Burlein JE, Benson LA, Bennett LT, Bansal GP, Young JF, Lee MH, Hatfull GF, Snapper SB, Barletta RG, Jacobs WR, Jr., Bloom BR. 1991. New use of BCG for recombinant vaccines. Nature 351:456-460.

3. Stephan J, Bender J, Wolschendorf F, Hoffmann C, Roth E, Mailander C, Engelhardt H, Niederweis M. 2005. The growth rate of *Mycobacterium smegmatis* depends on sufficient porin-mediated influx of nutrients. Mol Microbiol 58:714-730.
4. Wang Q, Boshoff HIM, Harrison JR, Ray PC, Green SR, Wyatt PG, Barry CEI. 2020. PE/PPE proteins mediate nutrient transport across the outer membrane of *Mycobacterium tuberculosis*. Science 367:1147-1151.
